# A Genome-Wide Systematic Analysis Reveals Different and Predictive Proliferation Expression Signatures of Cancerous vs. Non-Cancerous Cells

**DOI:** 10.1371/journal.pgen.1003806

**Published:** 2013-09-19

**Authors:** Yedael Y. Waldman, Tamar Geiger, Eytan Ruppin

**Affiliations:** 1 The Blavatnik School of Computer Science, Tel Aviv University, Tel Aviv, Israel; 2 The Sackler School of Medicine, Tel Aviv University, Tel Aviv, Israel; University of Washington, United States of America

## Abstract

Understanding cell proliferation mechanisms has been a long-lasting goal of the scientific community and specifically of cancer researchers. Previous genome-scale studies of cancer proliferation determinants have mainly relied on knockdown screens aimed to gauge their effects on cancer growth. This powerful approach has several limitations such as off-target effects, partial knockdown, and masking effects due to functional backups. Here we employ a complementary approach and assign each gene a cancer Proliferation Index (cPI) that quantifies the association between its expression levels and growth rate measurements across 60 cancer cell lines. Reassuringly, genes found essential in cancer gene knockdown screens exhibit significant positive cPI values, while tumor suppressors exhibit significant negative cPI values. Cell cycle, DNA replication, splicing and protein production related processes are positively associated with cancer proliferation, while cellular migration is negatively associated with it – in accordance with the well known “go or grow” dichotomy. A parallel analysis of genes' non-cancerous proliferation indices (nPI) across 224 lymphoblastoid cell lines reveals surprisingly marked differences between cancerous and non-cancerous proliferation. These differences highlight genes in the translation and spliceosome machineries as selective cancer proliferation-associated proteins. A cross species comparison reveals that cancer proliferation resembles that of microorganisms while non-cancerous proliferation does not. Furthermore, combining cancerous and non-cancerous proliferation signatures leads to enhanced prediction of patient outcome and gene essentiality in cancer. Overall, these results point to an inherent difference between cancerous and non-cancerous proliferation determinants, whose understanding may contribute to the future development of novel cancer-specific anti-proliferative drugs.

## Author Summary

One of the hallmarks of cancer is uncontrolled cellular proliferation, and therefore many anti-cancer drugs aim to disrupt cancer proliferation. However, some of these drugs (e.g., chemotherapeutic agents) affect normal proliferating cells as well, resulting in undesirable side effects. Understanding the differences between cancerous and non-cancerous proliferation can help us design new selective drugs that kill cancer cells without harming normal cells. In this work, we use genome scale gene expression and growth rate measurements across 60 cancer cell lines (NCI-60) to uncover genetic determinants of cancerous proliferation. In parallel, gene expression and growth rate measurements of non-cancerous cell lines allow us to uncover determinants of non-cancerous proliferation. Notably, we find marked differences between the cancerous and non-cancerous proliferation. The two proliferation signatures can be used jointly to enhance the prediction of patient outcome in cancer. Notably, we find that certain genes in the translation and spliceosome machineries are involved in cancerous proliferation but not in non-cancerous proliferation, highlighting them as putative selective anti-cancer drug targets.

## Introduction

Cancer is one of the leading causes of death worldwide and it is estimated that 12.7 million new cancer cases and 7.6 million cancer deaths occurred in 2008 [1]. One of the hallmarks of cancer is uncontrolled cellular proliferation [2], [3]. Understanding the determinants of cancer proliferation is an important task not only from a biological but also from a clinical stance – as the basis towards introducing new cancer therapies. Indeed, many chemotherapeutic agents target rapidly proliferating cells to fight cancer. However, these agents have additional detrimental effects on the non-cancerous, but proliferating tissues. In an attempt to find new anti-cancer drug targets, various studies in recent years used short hairpin RNA (shRNA) techniques and screened thousands of genes to find those that are essential for cancer growth and proliferation and are therefore putative targets for clinical intervention in cancer [4]–[7]. While this approach was shown to be powerful for the analysis of biological processes, shRNA screens have a variety of limitations such as off-target effects, partial knock down of the target genes and more [8]–[10]. Furthermore, as these are essentiality screens, they are less adequate to highlight genes involved in biological processes that have functional backups.

Here we set to explore the determinants of cancer proliferation by employing a complementary, computational approach, studying the association between gene expression and growth rate measurements on a set of cancer samples. Previous studies that analyzed cancer proliferation using expression data were limited in their extent, focusing on a small set of genes: Ross et al. [11] found that the expression of ribosomal proteins is highly correlated with doubling times of cancer cell lines and Gaur et al. [12] performed a similar analysis in order to find microRNAs whose expression is correlated with the doubling times of cancer cell lines. Others had defined and studied a set of proliferating genes based on prior knowledge from the literature (Gene Ontology (GO) annotation) [13]. Going beyond these earlier investigations, we present the first genome-wide analysis of cancer proliferation based on gene expression and growth rate measurements of 12690 genes across 60 cancer cell lines (the NCI-60 panel). We identify the genes and cellular processes that are related to cancer proliferation and determine whether their expression levels are positively or negatively associated with proliferation. By performing a similar analysis on non-cancerous proliferating human cells, we find marked differences between the genes whose expression is associated with cancerous and non-cancerous proliferation. This work lays the basis for future identification of selective anti-cancer drug targets.

## Results

### Computing a cancer Proliferation Index (cPI)

We focus on studying the NCI-60 panel, consisting of 60 different cancer cell lines originating from nine different tumor types [14]. This dataset contains growth rate measurements for all 60 cell lines and expression data for 12690 genes in these cell lines (Materials and Methods). We assign for each gene a measure, denoted as its cancer Proliferation Index (cPI). We define cPI as the non-parametric Spearman correlation between the expression of a gene and growth rate measurements across the NCI-60 panel. An alternative definition, which was also used in a previous work that analyzed proliferation determinants in yeast [15] is the slope of the regression line between the expression of a gene over the NCI-60 panel and growth rate measurements of these cell lines. The two measures are highly correlated (R = 0.89, P-value≪e-16, 12690 genes) and many of the results reported in the main text are also obtained using the regression slope as the cPI measure (Text S1). Table S1 provides the cPI values (by the two definitions) for 12690 genes analyzed in this study. Genes with high and positive (or low, negative) cPI values are positively (negatively) associated with cancer proliferation (see also Figures S1, S2, S3, S4).

### The functional significance of the cPI measure

**Figure 1:**
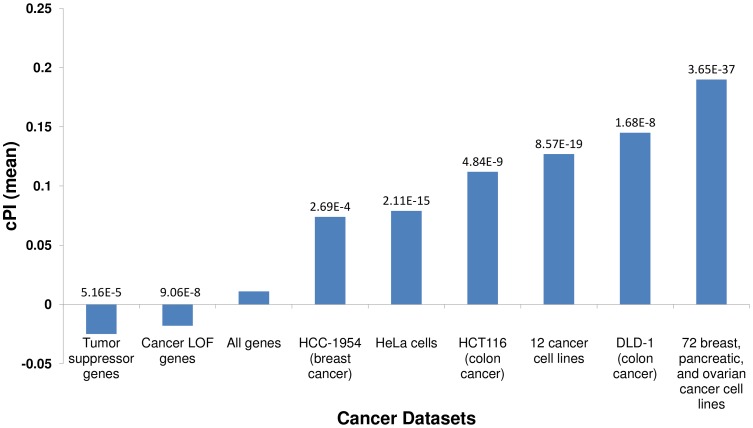
Essential genes and their cPI values. Using published shRNA screening data we defined sets of genes essential for cancer proliferation in different cancer cell lines (Materials and Methods). Each of these sets exhibits significantly high cPI values as compared to non-essential genes (Wilcoxon rank sum test). In contrast, tumor suppressor genes and genes with loss of function (LOF) mutations in various cancers show significantly lower cPI values. The mean cPI value of all genes is also depicted as a reference.

We first set out to compare the genes with significant (positive or negative) cPIs to other cancer related gene sets, to learn more about the potential overlap between these gene sets. First, in a comparison to several large-scale shRNA screens of cancer proliferation [4]–[7], we find that essential genes exhibit significantly higher cPI values relative to non-essential genes in all these screens. Second, we examined a set of 551 tumor suppressor genes [16] and a set of 1352 genes that were found to acquire loss of function mutations in various tumors and are therefore presumed to be enriched with tumor suppressors [17]. In both cases we find that these genes show significantly lower and negative cPI values as compared to other genes, testifying that their decreased functionality may indeed enhance cellular proliferation (Figure 1 and Figure S5). Third, we analyzed a known dataset of cancer-related genes (CancerGenes) [18], some that are tumor suppressors and some that are oncogenes. As genes belonging to this dataset may affect proliferation in opposite directions, we hypothesized that their absolute value of cPI (|cPI|) will be relatively high. Indeed, we find that the set of cancer genes exhibit higher |cPI| values as compared to other genes (P-value = 1.22e-5; 2735 genes; Materials and Methods). Taken together, these results demonstrate the relatedness of the cPI measure with important functional attributes of cancer-related genes, including their essentiality and their oncogenic role.

### Cellular processes playing a key role in cancer proliferation

Utilizing the cPI measure we set to examine which cellular processes are associated with cancer proliferation. We use the GO classification [19] and the set of pathways defined in the human metabolic model [20]. A full list of all GO terms and metabolic pathways whose members exhibit cPI values that are significantly different than the background distribution is provided in Tables S2 and S3. Here we review the key results.

Not surprisingly, cell cycle related processes (mitosis, DNA replication and packaging, telomere maintenance and more) that are vital for cellular proliferation exhibit significantly positive cPI values (after correcting for multiple hypotheses testing). In agreement with Ross et al. [11], we find that translation is positively correlated with proliferation in the NCI-60 panel, and more generally that protein production related processes (ranging from mRNA translation to protein localization in cell compartments) are positively correlated with proliferation. In addition, processes related to splicing also exhibit significant positive cPI values. This may be related to protein production in general, but may also have additional importance as splicing is known to play an important role in cancer [21], [22]. In addition, processes related to oxidative phosphorylation also show significant positive cPI values. On the other hand, many processes related to cell migration (cell migration, locomotion, cell adhesion) are negatively correlated with proliferation. This is in accordance with previous studies reporting a “go or grow” dichotomy in cancer denoting a negative correlation between invasive and proliferative phenotypes in tumors [23]–[27]. The inverse relation between migration and proliferation is further supported by additional datasets: first, we find that sets of genes that are related to cell migration exhibit significantly lower cPI values as compared to other genes: these include genes in the human integrin adhesome [28] (P-value = 5.54e-10) and genes whose silencing was previously shown to significantly damage cell migration [29] (empiric P-value = 3.96e-2). Second, we assigned for each gene a measure for its relatedness to cell migration based on PubMed papers (Materials and Methods). This measure is significantly negatively correlated with the cPI measure (R = −0.15, P-value = 1.08e-67; 12580 genes), in accordance with the “go or grow” dichotomy. In addition to the analysis on all 60 cell lines in the NCI-60 panel, we divided the cell lines into two groups based on growth rate. Overall, the results were very similar in both the slow and fast dividing cell lines (Tables S2 and S3). In addition, we repeated the analysis for each specific cancer type alone, obtaining tumor specific proliferation signatures. We find that genes associated with cholesterol metabolism have significantly negative cPI values in colon cancer, in accordance with studies showing that high density lipoprotein (HDL) cholesterol levels are inversely correlated with the risk of colon cancer [30]. Additional results are found in Tables S2 and S3. To evaluate the robustness of the results over different gene expression measurements, we repeated the main analyses using gene expression dataset of NCI-60 that was measured on a different platform, obtaining qualitatively similar results (Text S1 and Tables S2 and S3).

### Cancerous and non-cancerous proliferation signatures are markedly different

**Figure 2:**
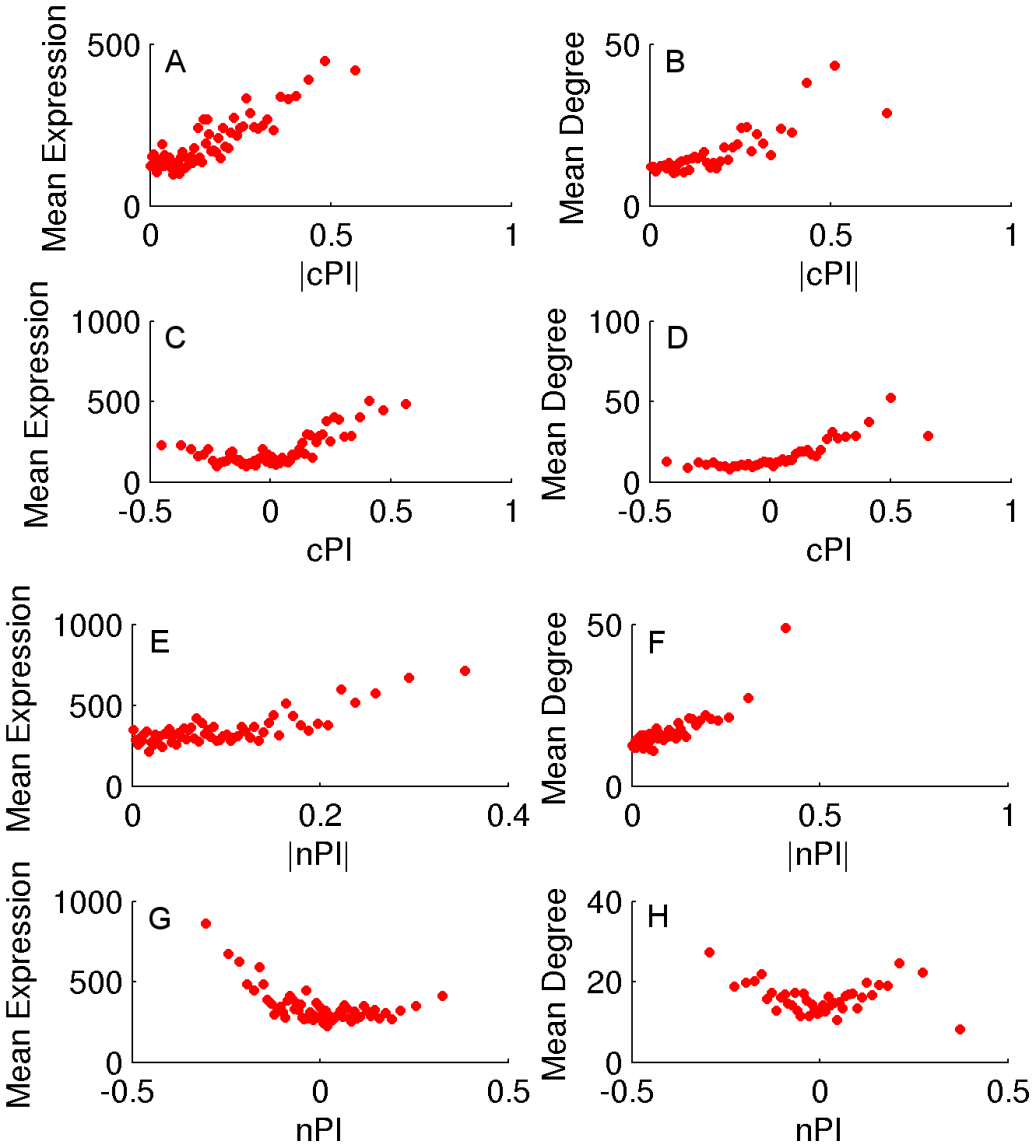
cPI and nPI vs. mean expression and degree in the human PPI network. Sorting the genes according to their PI measure (nPI, cPI or their absolute values) and binning them (200 genes in a bin), we calculate for each bin the average PI measure, mean expression and degree in the human PPI network. (A) |cPI| vs. mean expression in the NCI-60 panel (R = 0.23, P-value = 3.42e-146 and R = 0.81, P-value≪e-16 for the raw and binned data, respectively). (B) |cPI| vs. degree in the human PPI network (R = 0.13, P-value = 2.15e-32 and R = 0.82, P-value≪e-16 for the raw and binned data, respectively). (C) cPI vs. mean expression in the NCI-60 panel (R = 0.19, P-value = 1.89e-100 and R = 0.54, P-value = 6.76e-6 for the raw and binned data, respectively). (D) cPI vs. degree in the human PPI network (R = 0.16, P-value = 4.83e-48 and R = 0.86, P-value≪e-16 for the raw and binned data, respectively). (E) |nPI| vs. mean expression in 30 adult human tissues (R = 0.09, P-value = 3.44e-24 and R = 0.62, P-value = 2.37e-7 for the raw and binned data, respectively). (F) |nPI| vs. degree in the human PPI network (R = 0.09, P-value = 9.79e-15 and R = 0.81, P-value≪e-16 for the raw and binned data, respectively). (G) nPI vs. mean expression in 30 adult human tissues (R = −0.06, P-value = 4.12e-12 and R = −0.47, P-value = 1.40e-4 for the raw and binned data, respectively). (H) nPI vs. degree in the human PPI network (R = 0, P-value = 9.48e-1 and R = −0.07, P-value = 6.59e-1 for the raw and binned data, respectively).

We turn to study whether there are differences between the genes playing a key role in cancerous vs. non-cancerous proliferation. Obviously, if such differences can be identified, they can serve as a basis for selectively targeting cancer proliferation in the future. To identify the proliferation signature of non-cancerous proliferating cells we analyzed a dataset of HapMap samples that contains expression data and growth rates measurements for 224 lymphoblastoid cell lines from individuals from four different populations [31]. We assign a non-cancerous PI (nPI) measure for 12690 genes in this dataset (Materials and Methods and Table S1). In our analysis we used all 224 cell lines, but the results remain qualitatively similar also when focusing on samples from different populations or specific gender (Materials and Methods, Text S1 and Tables S2 and S3). Comparing the cPI and nPI measures of genes, we find that cancerous and non-cancerous proliferation are markedly different. First, the correlation between the two PI measures is relatively low (R = 0.07, P-value≪e-16). The differences are also reflected by the relation of these two measures to other genomic measures. Both |cPI| and |nPI| are positively associated with mean expression (in cancerous and non-cancerous tissues, respectively), as well as with the degree of the gene's product in the human protein-protein interaction (PPI) network. Nevertheless, when we turn to cPI and nPI (instead of |cPI| and |nPI|) we observe different trends: while cPI is positively correlated with both measures (mean expression and degree in human PPI), the latter measures are not positively correlated with nPI and we even find slightly negative association with expression in normal tissues (Figures 2 and S6).

An analysis of GO terms points to key functional differences between non-cancerous and cancerous proliferation on the process level (Tables S2 and S3). Reassuringly, cell cycle processes exhibit significantly positive PI values in both proliferation types. However, protein production related processes exhibit an opposite behavior - positive correlation with cancerous proliferation but negative correlation with non-cancerous proliferation. The negative nPI values of protein production associated genes are also supported by a previous study on non-cancerous immortalized human keratinocytes that showed decrease in protein production as proliferation increases [32]. Overall, in cancer cells the expression of the translation machinery but also the expression of macromolecule and protein catabolic processes increases as proliferation increases. In contrast, in non-cancerous cells we observe a decrease in both the expression of translation and degradation machineries as proliferation increases, probably allowing for a more efficient usage of proteins with less turnover associated energy costs. Another mechanism accounting for the decrease in translation in non-cancerous proliferation may be a reduction in cell size [32].

**Figure 3:**
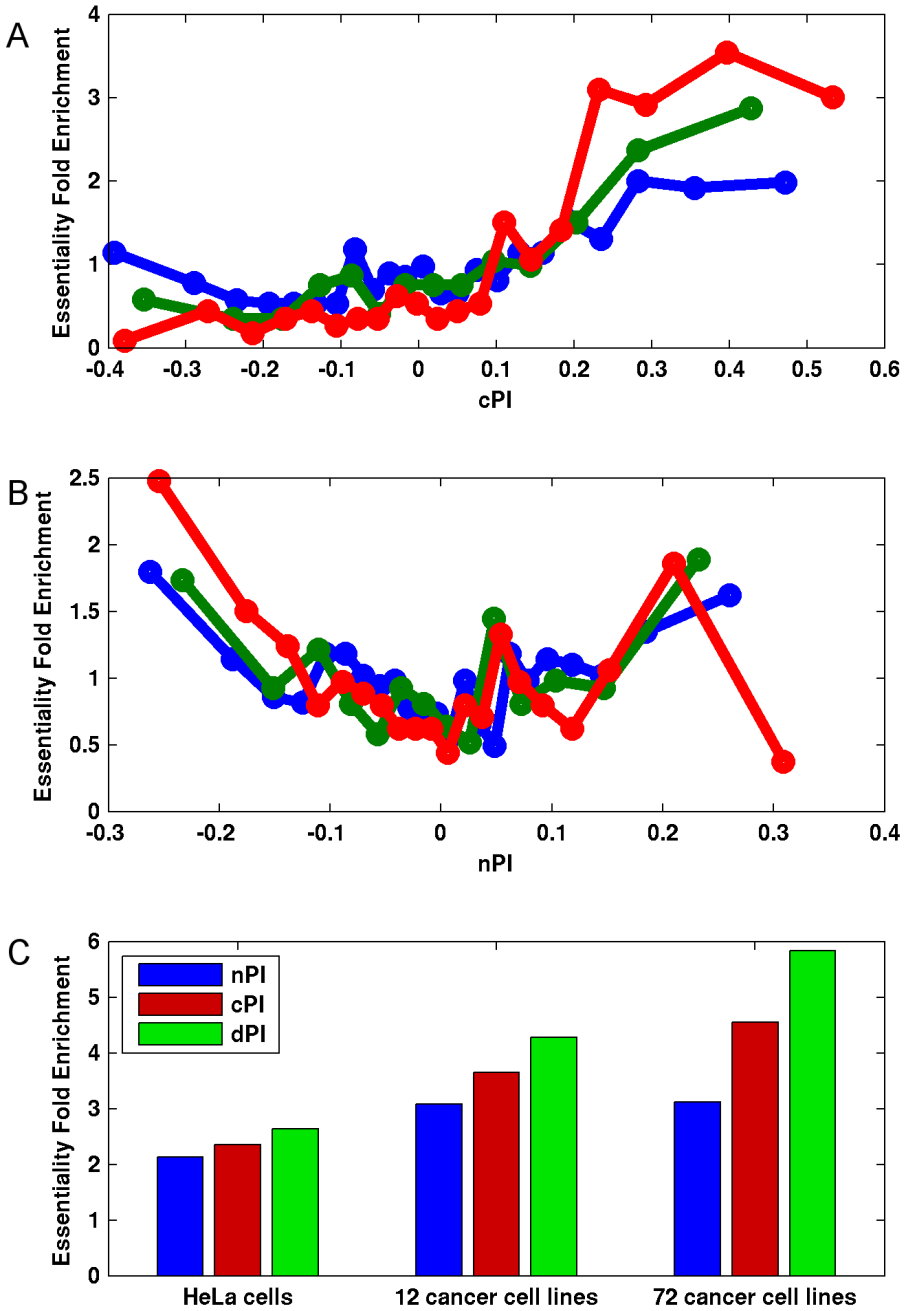
Differential proliferation signatures and cancer gene essentiality. We grouped the genes into bins (200 genes in each bin) according to their (A) cPI and (B) nPI values and measured for each bin the mean measure (cPI or nPI) and the enrichment of the genes in the bin in essential genes in HeLa cells (blue), 12 cancer cell lines (green) and 72 breast, pancreatic, and ovarian cancer cell lines (red). (C) Focusing on a set of 3210 genes with positive cPI and negative nPI values, we defined the top 200 genes for each measure (lowest nPI, highest cPI, highest dPI, correspondingly) and find that dPI shows the highest enrichment in all datasets. Enrichment is significant in all cases (hypergeomteric P-value<4e-3).

Notably, we find marked differences between cPI and nPI measures also with respect to gene essentiality in cancer. As expected, the likelihood of a gene to be essential to cancer proliferation increases as cPI increases (Figures 3A and S7A). Yet, nPI exhibits a different behavior: genes with both highly positive and highly negative nPI values are enriched with essential genes, with some more enrichment for those with negative values (Figures 3B and S7B). Focusing on a set of 3210 genes that exhibits differential proliferation (genes with positive cPI and negative nPI values) we define a new measure, the differential Proliferation Index (dPI). dPI combines the two other measures (nPI and cPI) to reflect differential proliferation and assigns higher values to genes with a more differential proliferation signature (i.e., genes with high cPI values and low nPI values; Materials and Methods). We find that this joint measure better predicts cancer gene essentiality as compared to each of the other two measures alone (Figures 3C and S7C). This finding is highly intriguing, as it appears that information on the association of a gene to proliferation in non-cancerous cell lines can give us additional information on its association to essentiality in cancer.

Furthermore, the usage of dPI may help us find genes that might serve as selective drug targets – i.e., genes that are more involved in cancer proliferation than in non-cancerous proliferation. Analysis of the 200 genes with the highest dPI values shows them to be enriched in genes belonging to the translation (mainly ribosomal proteins) and spliceosome machineries (Table S4). This is in accordance with previous studies suggesting these biological processes as targets in cancer therapy (Discussion).

### nPI, cPI and dPI signatures predict growth rates

**Figure 4:**
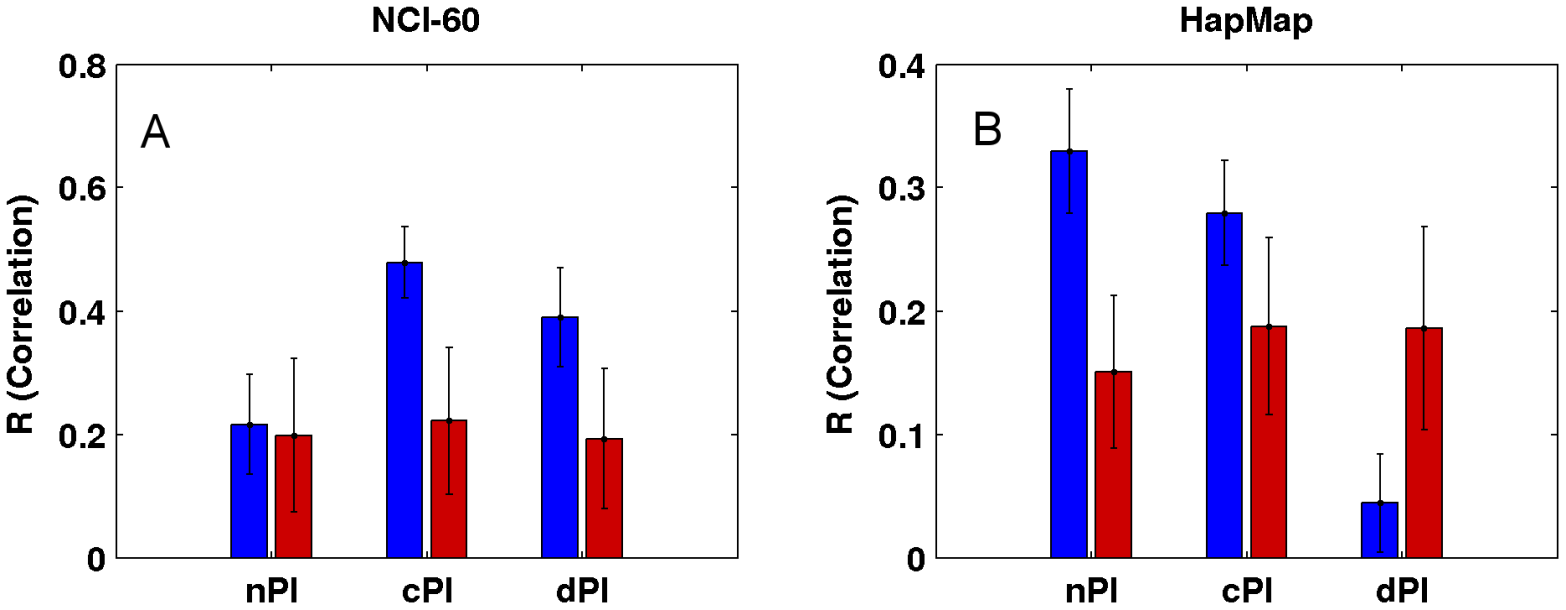
Growth rate predictions of the (A) NCI-60 and (B) HapMap panels. For each signature (nPI, cPI, dPI) we report the correlation between the predicted and measured growth rate in the panels. The mean R correlation (in blue) is compared to the mean R correlation of equal size random sets of genes (in red; Materials and Methods).

Not surprisingly, and as one could have expected, our measures (nPI, cPI and dPI) can also predict growth rates in the NCI-60 and HapMap panels. Thus, we defined for each of the three measures a signature which is the set of genes with significant values of this measure (Materials and Methods). We then trained a linear regression based predictor on these signatures to predict growth rates (Materials and Methods). Reassuringly, we find that the signatures of the cancer related measures can successfully predict growth rates in the NCI-60 panel, significantly better than equal size random sets of genes (empiric P-value = 3.96e-30 and empiric P-value = 2.88e-25 for cPI and dPI, respectively), while the nPI signature does not achieve significantly better prediction compared to that obtained using random signatures (empiric P-value = 0.31; Figure 4 and Figure S8). Turning to the HapMap panel, we find that the non-cancerous signature nPI achieves the best results (empiric P-value = 5.71e-33). cPI also exhibit significant predictions accuracy, but to a lesser extent (empiric P-value = 7.49e-19). Interestingly, not only the dPI signature does not outperform random gene sets but it even shows significantly poorer prediction capabilities (empiric P-value = 7.41e-26).

### cPI and dPI signatures successfully predict survival outcome analyzing clinical samples

**Figure 5:**
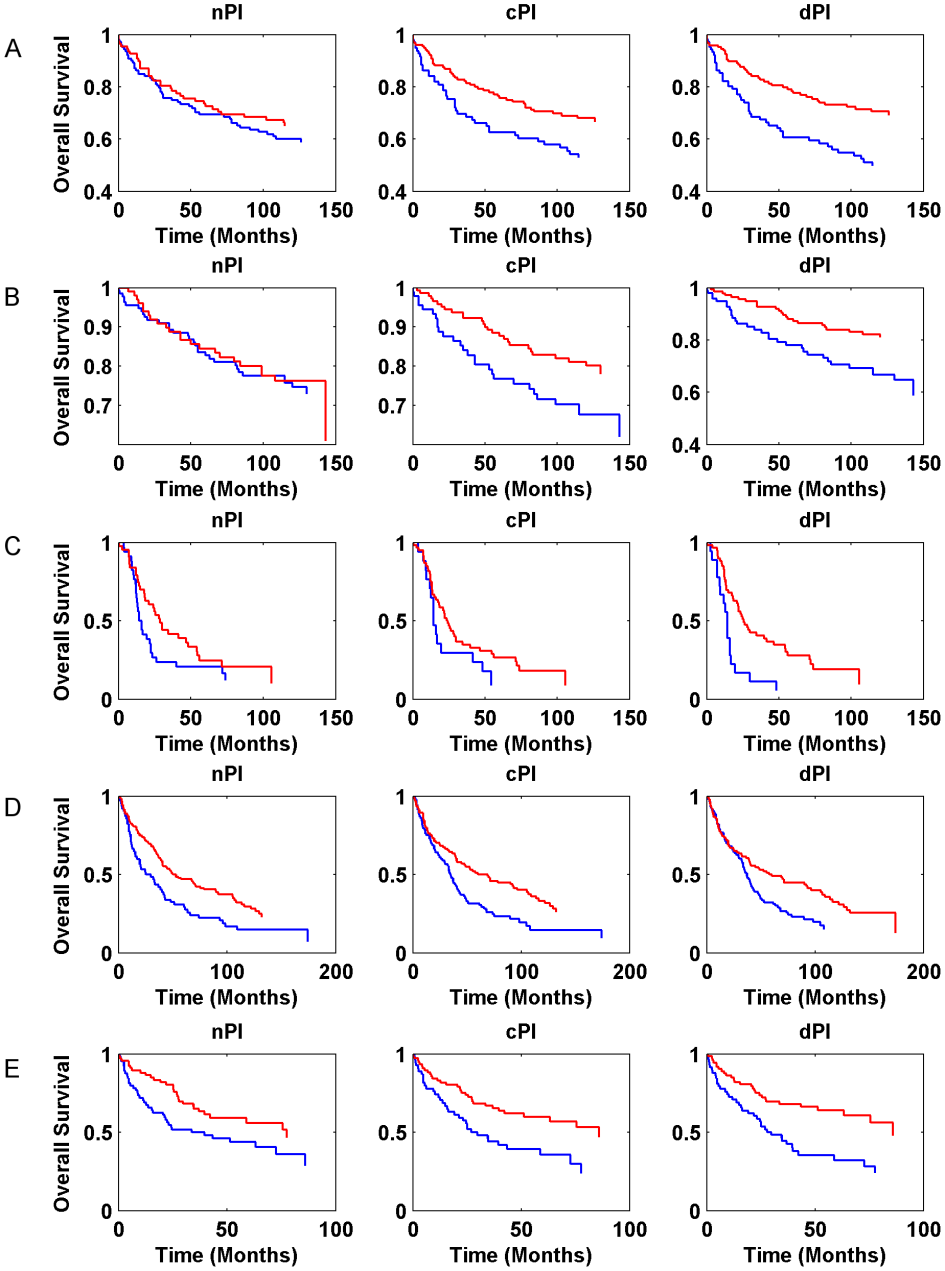
Kaplan-Meier curves for nPI, cPI and dPI signatures in various cancer datasets. (A) breast cancer (Ivshina et al., 249 samples); (B) breast cancer (Miller et al., 236 samples); (C) glioma (Phillips et al., 77 samples); (D) NSCLC (Botling et al., 196 samples). (E) CLL (Chuang et al., 130 samples). The blue and red curves represent lowly and highly predicted proliferating samples, respectively. Additional information is found in Table S5.

Gene expression signatures can classify tumor classes and predict patient survival [33]–[35]. Moreover, in various cases, expression signatures for cancer prognosis contain many proliferation associated genes and thus much of the prognostic power of these signatures is related to proliferation [13], [36]–[39]. In this context, we checked whether the signatures of the different measures (nPI, cPI and dPI) have a prognostic power to predict cancer patient survival. Similar to the analyses described above, we trained a linear regression predictor for growth rate on the NCI-60 (for cPI and dPI) and HapMap (for nPI) panels. Next, we applied this predictor to classify clinical samples based on their predicted growth rates to see whether such classification is associated with prognosis (Methods). For that purpose we used information on 888 patients with four different cancer types: two breast cancer datasets (485 patients), a non-small cell lung carcinoma (NSCLC) dataset (196 patients), a glioma dataset (77 patients), and a chronic lymphocytic leukemia (CLL) dataset (130 samples). Figure 5 (and Figure S9) and Table S5 show the predictive power of the different signatures (nPI, cPI, dPI) on these datasets. Overall, we find that the signatures of cancer associated measures (cPI and dPI) achieve better results than the non-cancerous associated measure (nPI). Specifically, the dPI signature achieves significant results (logrank P-value<0.05) in all five datasets, the cPI signature achieves significant results in four of the datasets while the nPI signature achieves significant results in two of the datasets. Cox regression analyses shows that in some but not all cases, there is added value for the signatures beyond clinical features such as grade, age, estrogen receptor status, lymph node status and more (Table S5). Taken together, these results demonstrate the predictive power of both cPI and dPI signatures (beyond that of nPI) in various cancer types.

### Cancer proliferation resembles that of microorganisms while non-cancerous proliferation does not

Lastly, and after showing that cancerous and non-cancerous proliferation signatures are markedly different, we compare their relation to the proliferation signatures of microorganisms. Two previous studies measured gene expression under various growth rates in the *Saccharomyces cerevisiae* yeast [15] and in the *Lactococcus lactis* bacteria [40]. Both studies found that the expression of translation related genes, and specifically of ribosomal proteins, is upregulated as growth rate increases, in accordance with cancerous proliferation, and opposite to non-cancerous proliferation. Furthermore, Brauer et al. [15] also calculated the slope between expression and growth rate in yeast, analogues to our PI measures. We find a significant correlation between cPI and yeast PI for 1659 orthologous genes (R = 0.19, P = 6.12e-15), whereas the correlation between nPI and yeast PI is much weaker (R = 0.08, P = 4.47e-4). Notably, the correlation between nPI and cPI on those 1659 genes (R = 0.11, P-value = 7.68e-6) is weaker than the correlation between cPI and yeast PI measure. In addition, we looked on sets of genes that were found to be essential in two different yeast species. Indeed, we find that the cPI of their human orthologous genes is significantly higher as compared to other genes (mean cPI = 0.18, P-value = 1.09e-86 and mean cPI = 0.18, P-value = 2.40e-100 for *Saccharomyces cerevisiae* (572 genes) and *Schizosaccharomyces pombe* (709 genes) essential genes, respectively; Wilcoxon test). Similar analysis also reveals that these genes tend to have higher nPI values as well, but to much smaller extent (mean nPI = 0.02, P-value = 2.73e-6 and mean nPI = 0.02, P-value = 6.90e-7 for *Saccharomyces cerevisiae* and *Schizosaccharomyces pombe* essential genes, respectively; Wilcoxon test). These results suggest that cancer and microorganisms' proliferation are quite similar and closer to each other and are both quite distinct from non-cancerous proliferation. This may testify that cancer cells utilize cell programs that are characteristic of unicellular organisms, in contrast to the multi-cellular cooperative programs of normal healthy human cells. Interestingly, an array of other similarities between cancer and microorganisms were already highlighted by previous studies [41].

## Discussion

In the current work we identify gene signatures of cancer proliferation, based on a large scale analysis of gene expression and growth rate measurements of 12690 genes across the NCI-60 panel. The results show that the emerging cPI signature is highly relevant, having clear functional correlates. Our analysis uncovers genes and cellular processes that are associated with proliferation, either positively or negatively, in various cancers.

Our non-cancerous analysis reveals marked differences between cancerous and non-cancerous proliferation. Currently, we lack additional datasets that include both expression and growth rate measurements of non-cancerous cells or cell lines to further support and generalize these results. Recently, Im et al. [42] calculated growth rates for HapMap samples based on a different experimental method and a model termed mixed effects model averaging (MEM). They report a correlation of R = 0.30 between their MEM growth rates and the growth rates directly measured by Choy et al. [31] (and used in the current study). In their paper, Im et al. also report a measure that is analogous to our nPI measure, and is based on the association between MEM values and expression levels on 176 HapMap samples measured in their lab (136 of them also appear in our dataset). We used their measure (termed here nPI-MEM) to see if it can support our results. Overall, the results obtained by nPI-MEM are weaker than those obtained by nPI. Several reasons may account for this. First, gene expression of the same sample may very across time and as opposed to Choy et al., growth rate and expression measurements of each cell line were not necessarily taken in the same time. In addition, Choy et al. used direct measurements of cell lines' growth rate while Im et al. used an estimator which is based on a model and also on indirect measurements of growth rate. Nevertheless and given all these limitations, we still find that the nPI-MEM is more similar to nPI than to cPI. Thus, we find that nPI-MEM shows significant correlation to nPI (R = 0.16, P-value = 1.32e-43; 7443 genes). The correlation between nPI-MEM and cPI is significant but weaker (R = 0.065, P-value = 1.80e-8). When focusing on a subset of 2187 genes whom Im et al. defined as associated (either positively or negatively) with proliferation, the correlation between nPI and nPI-MEM becomes much stronger and much higher (R = 0.294, P-value = 1.58e-44) as compared to the correlation between cPI and nPI-MEM on the same set of genes (R = 0.118, P-value = 3.73e-8). Additional and detailed results are described in Text S1 and in Table S6.

In addition to the support for the nPI measure from the independent measurements of Im et al., we find additional support by the fact that combining the two proliferation signatures (cPI and nPI) has an added value in cancer research: the differential proliferation measure dPI can better predict a likelihood of a gene to be essential to cancer proliferation (Figure 3) as compared to each of the proliferation measures (cPI and nPI) alone and also has predictive power in patient prognosis (Figure 5).

Compared with genome-wide knockdown genetic screens, our approach is less sensitive to the potential masking by backups of a gene's contribution to proliferation. Nevertheless, it obviously has its own limitations. Similar to other high-throughput techniques, there is experimental noise in the measurements. Obviously, a correlation between expression and growth rate does not necessarily imply causality and may reflect indirect associations. The two different approaches, which exhibit a significant overlap in their predictions, are complementary, and together lay the foundation for small scale studies to validate specific emerging predictions of interest.

Specifically, we find biological processes and machineries that are enriched with genes with high dPI values that might be used as putative selective anti-cancer drug targets. These include members of the translation (mainly ribosomal proteins) and the spliceosome machineries (Table S4). Notably, previous studies already suggested these machineries as targets in cancer therapy. Thus, targeting translation initiation [43], [44] and elongation [45] cause a therapeutic response in various tumors. Although the ribosome plays an important role also in non-cancerous cells, extensive reduction in ribosomal activity may be more tolerated in non-cancerous cell proliferation but not in cancer cells where there is a greater demand for it [46]. Indeed, targeting ribosomal RNA genes selectively kills B lymphoma cells while maintaining a viable wild-type B cell population [47]. Furthermore, FDA recently approved the ribosome targeting drug SYNRIBO (omacetaxine mepesuccinate), for specific cases of chronic myeloid leukemia (CML). In addition, various compounds that inhibit cancer cells growth target the spliceosome [48].

The results presented here lay a genome-scale view for future studies aimed at teasing apart the differences between non-cancerous and cancerous proliferation, paving the way towards novel selective cancer therapeutics.

## Materials and Methods

### PI measure

The PI of a gene reflects the association between its expression levels and growth rates measurements across a set of samples. In the main text we use the non-parametric Spearman correlation between the expression levels and growth rates as a measure for PI while in Text S1 we perform a similar analysis using the slope of the regression line between expression levels and growth rates. In this case (the slope based measure) we normalized the PI measure by dividing it with the median absolute PI value of this dataset. We used a log2 transformation of the expression data. We assigned a P-value for the PI measure of each gene based on the significance of the correlation (Spearman P-value) or the regression line (F-statistic P-value, as calculated by the MATLAB ‘regress’ function).

For cPI we used the NCI-60 panel (60 samples). Gene expression data for the NCI-60 panel was downloaded from Gene Expression Omnibus [49], GSE5846 series, and is based on [50]. Doubling times for the NCI-60 cell lines were downloaded from the website of the Developmental Therapeutics Program (DTP) at NCI/NIH (http://dtp.nci.nih.gov/docs/misc/common_files/cell_list.html; accessed on August 2013). For tumor specific analysis we used a subset of samples from the NCI-60 of the specific tumor type. We ignored prostate cancer as the NCI-60 has only 2 samples from this origin. To evaluate the robustness of the expression measurements, we also repeated the analysis using gene expression from a different platform (GSE29288), obtaining similar results (Text S1 and Tables S2 and S3).

For nPI we used the HapMap panel. Gene expression and growth rate measurements for 224 proliferating lymphoblastoid cell lines of the HapMap panel were taken from [31] and were collected from four different populations: Utah residents with Northern and Western European ancestry (CEU; 56 samples), Han Chinese in Beijing, China (CHB; 43 samples), Japanese in Tokyo, Japan (JPT; 43 samples) and Yoruba from Ibadan, Nigeria (YRI; 82 samples). A previous paper [51] suggested that some mix-up might have occurred in the labels of the gene expression samples of a few of the HapMap cell lines. To examine the robustness of the results we also repeated our analysis for each population alone (as the mix-up was found mainly in the Asian populations). In addition, nPI values remain very similar if the mix-up samples are excluded or re-classified according to [51] (R>0.97, P-value≪e-16 for nPI based on correlation or slope).

In addition to the nPI and cPI measures, we defined a new measure, dPI (differential Proliferation Index), for the set of genes exhibiting differential proliferation (genes with positive cPI but negative nPI values). Specifically, let 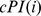 and 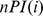 be the cPI and nPI values, respectively, of gene *i*. We define 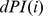, the dPI value of gene *i*, as:
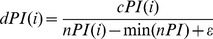
Where 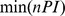 is the minimal nPI value among all genes. The denominator (which is always positive as 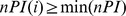) becomes smaller as 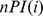 decrease and hence the value of 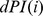 increases. We added 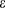 (set to be 0.01) to avoid division in zero.

For yeast (*S. cerevisiae*) data, we used the slope of the regression values between expression and growth rata measurements across 36 samples (six media and six growth rates in each media), as provided by Brauer et al. [15] and focused on 1659 genes whose human orthologs had PI values as well. Orthology to human genes was based on inParanoid 7.0 database [52].

### Functional enrichment analysis

Given a set of genes (either GO term or metabolic pathway), we compared the cPI (or nPI) distribution of the members of this set to that of all genes in the panel. We evaluated the difference between the two distributions using the Wilcoxon rank sum test. GO [19] annotations were downloaded from www.geneontology.org on March 2012. We included all annotations except for those inferred from electronic annotation (IEA). Classification of genes into metabolic pathways was based on the human metabolic model [20].

GO Analysis was done for each ontology (“molecular function,” “biological process,” and “cellular component”) separately and we ignored terms with less than 25 genes (and metabolic pathways with less then 5 genes). To address the problem of multiple hypotheses we used Bonferroni correction.

For the enrichment analysis of the 200 selective genes (genes with highest dPI values) we used DAVID Bioinformatics enrichment tools [53] (functional annotation).

### Predicting growth rates

Growth rate prediction was based on lasso regression [54] on the NCI-60 and HapMap panels. For each measure (nPI, cPI, dPI) we focused on the genes with significant values (after using Bonferroni correction) as determined by their P-values. As dPI is a combined measure, the P-values for the dPI genes were defined as the cPI P-values for these genes. We then used the expression levels of these genes in the input samples to predict their growth rates. We divided each panel (NCI-60 and HapMap) into training and test sets. The test set was composed of two samples and the other samples were in the training set. We trained a lasso regression predictor on the training set (λ = e-3, using a 5-fold cross validation procedure on this set) to predict the growth rates of the samples in the test set. We repeated this procedure (i.e., cross-validation) until growth rates were predicted for all samples in the panel and we could evaluate the correlation between the predicted and actual growth rates. We repeated this process 100 times (each time taking random divisions of train and test sets). We evaluated the performance of the different signatures (nPI, cPI and dPI) by comparing (using a Wilcoxon rank sum test) the correlations values obtained by the signatures to that obtained by performing the same analysis for equal size random sets of genes. Each of the measure signatures (nPI, cPI and dPI) contains a different number of genes (see Table S5) and therefore we used different random sets for the different signatures.

### Predicting survival

Given a set of clinical samples, we aim to separate the samples into two groups based on expression data, and see whether these two groups have significantly different survival outcome. The separation of the samples into two groups is based on predicting their growth rates and then clustering them into lowly and highly proliferating samples (using *K*-means clustering, *k* = 2). The significance of the separation was evaluated by a logrank test, as described previously [55].

We predicted the growth rates of each sample similar to the way we predicted growth rates in the NCI-60 and HapMap panels (as described above). Specifically, we trained for each signature a lasso regression predictor on a panel (HapMap panel for the nPI signature, NCI-60 panel for the cPI and dPI signatures). We used a 5-fold cross validation procedure, and chose the λ value that minimizes mean squared error on this panel (using the MATLAB ‘lasso’ function). Table S5 exhibits the weights for the different gene signatures. We then applied these predictors (one predictor for each signature) to a clinical dataset and predicted for each sample its growth rate. In cases where not all 12690 genes used in the primary analysis were present in the clinical gene expression data, the nPI, cPI and dPI signatures were taken from the genes with significant nPI, cPI and dPI values respectively that were also present in the clinical gene expression data. In both analyses (predicting growth rates and predicting survival), we used a log2 transformation on the expression data.

The datasets included in our analysis are: Miller et al. (breast cancer, 236 samples) [56], Ivshina et al. (breast cancer, 249 samples) [57], Phillips et al. (glioma, 77 samples) [58], Chuang et al., (CLL, 130 samples) [59], and Botling et al. (NSCLC, 196 samples) [60].

### Essential genes for cancer proliferation

Lists of essential genes for cancer proliferation were taken from various short hairpin RNA (shRNA) screening: (a) 12 cancer cell lines [6] (we used the “commonly essential” set); (b) 72 breast, pancreatic, and ovarian cancer cell lines [7] (we used the set of essential genes in all three tumor types); (c) DLD-1 (colon cancer) cell line [5]; (d) HCT-116 (colon cancer) cell line [5]; (e) HCC-1954 (breast cancer) cell line [5]; (f) HeLa cells [4] (a screen for cell division related genes).

The enrichment of a certain group of genes with essential genes is defined by the fraction of essential genes in this group divided by the fraction of essential genes in a reference group. When available, the reference group was the set of all genes that were screened for essentiality (and have PI measure) and otherwise the reference group was the set of all genes with PI measure. We used hypergeometric test to evaluate the significance of the enrichment. Similarly, we evaluated the PI measure of certain group of genes (e.g., the cPI values of essential genes) by comparing their values to that of a reference group (as explained above) using the Wilcoxon rank sum test.

### Yeast essential genes

A list of essential genes in yeasts was taken from Kim et al. [61] for *Schizosaccharomyces pombe* and from Winzeler et al. [62] for *Saccharomyces cerevisiae*. Orthology to human genes is based on inParanoid 7.0 database [52].

### Cancer related gene sets

A list of genes with loss-of functions mutations in cancers was taken from [17]. In addition, we used a list of genes based on CancerGenes, a database of cancer related genes [18]. A list of tumor suppressor genes was taken from [16].

### Cell migration analysis

We used two datasets of genes related to cell migration: (a) genes in the human integrin adhesome [28] and (b) a list of genes whose silencing damages cell migration [29]. We compared the cPI distribution of the genes in human integrin adhesome (using Wilcoxon test) for significance. As the list from [29] is relatively smaller (24 genes have cPI values), we empirically assessed the significance of their lower cPI values, by comparing the mean cPI of this set to 100,000 random equal size gene sets from their screening dataset. In addition, we quantified the association between a gene and cell migration using PubMed [63]. For each gene we counted the number of papers associated with it and with the MeSH term “cell migration”. Association between papers and genes was based on the gene2pubmed file (ftp://ftp.ncbi.nih.gov/gene/DATA/; accessed August 2012).

### Expression in normal tissues and human PPI data

Expression in 30 non-cancerous adult tissues and degree in the human PPI were calculated as described in [64].

All the correlations reported in this work are the non-parametric Spearman correlation.

## Supporting Information

Figure S1 Expression vs. doubling times in the NCI-60 panel for genes with extreme cPI values (correlation based). The genes in the top two panels have cPI value above the 99.9 percentile (highest cPI values) while the genes in the bottom two panels have cPI values below the 0.1 percentile (lowest cPI values). The Gene ID (Entrez) for each gene is written above the panel.
(TIFF)

Figure S2 Expression vs. growth rate in the HapMap panel for genes with extreme nPI values (correlation based). The genes in the top two panels have cPI value above the 99.9 percentile (highest nPI values) while the genes in the bottom two panels have nPI values below the 0.1 percentile (lowest nPI values). The Gene ID (Entrez) for each gene is written above the panel.
(TIFF)

Figure S3 Expression vs. doubling times in the NCI-60 panel for genes with extreme cPI values (slope based). The genes in the top two panels have cPI value above the 99.9 percentile (highest cPI values) while the genes in the bottom two panels have cPI values below the 0.1 percentile (lowest cPI values). The Gene ID (Entrez) for each gene is written above the panel.
(TIFF)

Figure S4 Expression vs. growth rate in the HapMap panel for genes with extreme nPI values (slope based). The genes in the top two panels have cPI value above the 99.9 percentile (highest nPI values) while the genes in the bottom two panels have nPI values below the 0.1 percentile (lowest nPI values). The Gene ID (Entrez) for each gene is written above the panel.
(TIFF)

Figure S5 Essential genes and their cPI values (slope based). Using published shRNA screening data we defined sets of genes essential for cancer proliferation in different cancer cell lines (Materials and Methods). Each of these sets exhibits significantly high cPI values as compared to non-essential genes (Wilcoxon rank sum test). In contrast, genes with loss of function (LOF) mutations in various cancers show significantly lower cPI values. The mean cPI value of all genes is also depicted as a reference.
(TIF)

Figure S6 cPI and nPI vs. mean expression and degree in the human PPI network (cPI and nPI are slope-based). Sorting the genes according to their PI measure (nPI, cPI or their absolute values) and binning them (200 genes in a bin), we calculate for each bin the average PI measure, mean expression and degree in the human PPI network. (A) |cPI| vs. mean expression in the NCI-60 panel (R = −0.11, P-value = 2.53e-37 and R = 0.39, P-value = 1.59e-3 for the raw and binned data, respectively). (B) |cPI| vs. degree in the human PPI network (R = 0.04, P-value = 9.54e-5 and R = 0.72, P-value = 3.35e-7 for the raw and binned data, respectively). (C) cPI vs. mean expression in the NCI-60 panel (R = 0.24, P-value≪e-16 and R = 0.79, P-value≪e-16 for the raw and binned data, respectively). (D) cPI vs. degree in the human PPI network (R = 0.18, P-value = 1.32e-61 and R = 0.95, P-value≪e-16 for the raw and binned data, respectively). (E) |nPI| vs. mean expression in 30 adult human tissues (R = 0.01, P-value = 2.11e-1 and R = 0.38, P-value = 2.94e-3 for the raw and binned data, respectively). (F) |nPI| vs. degree in the human PPI network (R = 0.03, P-value = 1.00e-2 and R = 0.20, P-value = 2.15e-1 for the raw and binned data, respectively). (G) nPI vs. mean expression in 30 adult human tissues (R = −0.19, P-value = 3.89e-98 and R = −0.82, P-value≪e-16 for the raw and binned data, respectively). (H) nPI vs. degree in the human PPI network (R = 0.07, P-value = 1.05e-11 and R = −0.80, P-value = 5.15e-9 for the raw and binned data, respectively).
(TIFF)

Figure S7 Differential proliferation signatures and cancer gene essentiality. We grouped the genes into bins (200 genes in each bin) according to their (A) cPI and (B) nPI values and measured for each bin the mean measure (cPI or nPI) and the enrichment of the genes in the bin in essential genes in HeLa cells (blue), 12 cancer cell lines (green) and 72 breast, pancreatic, and ovarian cancer cell lines (red). (C) Focusing on a set of 3331 genes with positive cPI and negative nPI values, we defined the top 200 genes for each measure (lowest nPI, highest cPI, highest dPI, correspondingly) and find that dPI shows the highest enrichment in all datasets. Enrichment is significant in all cases (hypergeomteric P-value<e-5). nPI, cPI and dPI measures are slope-based (see main text).
(TIFF)

Figure S8 Growth rate predictions of the (A) NCI-60 and (B) HapMap panels. For each signature (nPI, cPI, dPI [slope based]) we compared between the predicted and measured growth rate in the panels. The mean R correlation is presented here for each measure (in blue), in joint with the mean R correlation of equal size random sets of genes (in red). The three measures (nPI, cPI, dPI) are slope-based.
(TIFF)

Figure S9 Kaplan-Meier curves for nPI, cPI and dPI signatures (slope-based) in various cancer datasets. (A) breast cancer (Ivshina et al., 249 samples); (B) breast cancer (Miller et al., 236 samples); (C) glioma (Phillips et al., 77 samples); (D) NSCLC (Botling et al., 196 samples). (E) CLL (Chuang et al., 130 samples). The blue and red curves represent lowly and highly predicted proliferating samples, respectively. Additional information is found in Table S5.
(TIF)

Table S1 nPI, cPI and dPI values for the genes analyzed in this study.
(XLS)

Table S2 Functional analysis of cPI and nPI values (correlation based).
(XLS)

Table S3 Functional analysis of cPI and nPI values (slope based).
(XLS)

Table S4 Gene associated with cancer specific proliferation (top 200 genes with highest dPI values).
(XLS)

Table S5 Survival prediction in five cancer data sets.
(XLS)

Table S6 nPI-MEM analysis.
(XLS)

Text S1 Supplementary results (PI measure – correlation vs. slope, replication of the NCI-60 results on a different expression dataset, nPI and nPI-MEM).
(PDF)
