# Supplementary figures and images for: A Genome-Wide Systematic Analysis Reveals Different and Predictive Proliferation Expression Signatures of Cancerous vs. Non-Cancerous Cells
Source: PLoS Genet. 2013 Sep 19;9(9):e1003806. doi: 10.1371/journal.pgen.1003806 (PMC3778010; doi:10.1371/journal.pgen.1003806)

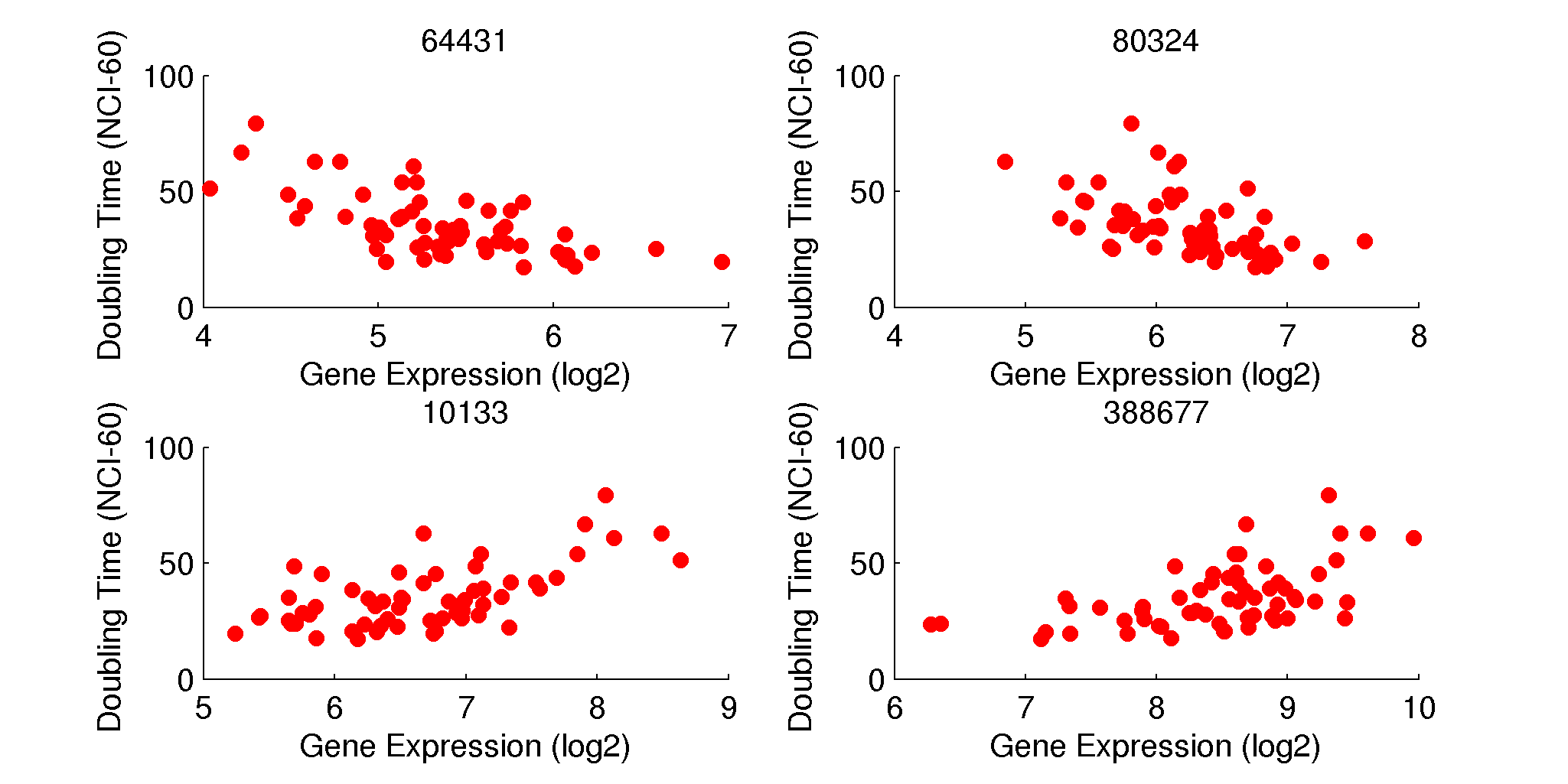

Supplement: Figure S1 — Expression vs. doubling times in the NCI-60 panel for genes with extreme cPI values (correlation based). The genes in the top two panels have cPI value above the 99.9 percentile (highest cPI values) while the genes in the bottom two panels have cPI values below the 0.1 percentile (lowest cPI values). The Gene ID (Entrez) for each gene is written above the panel. (TIFF) [file pgen.1003806.s001.tiff]

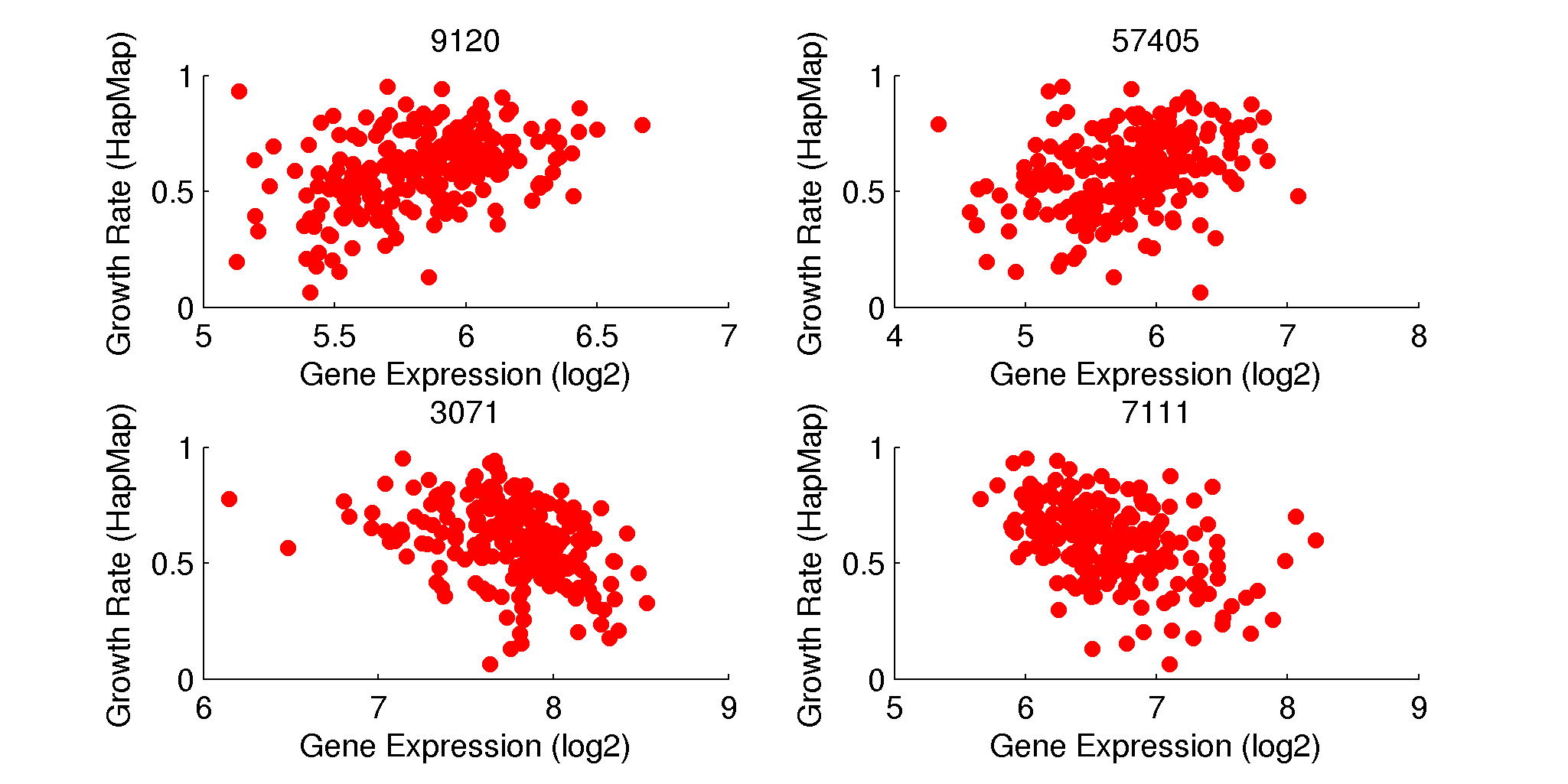

Supplement: Figure S2 — Expression vs. growth rate in the HapMap panel for genes with extreme nPI values (correlation based). The genes in the top two panels have cPI value above the 99.9 percentile (highest nPI values) while the genes in the bottom two panels have nPI values below the 0.1 percentile (lowest nPI values). The Gene ID (Entrez) for each gene is written above the panel. (TIFF) [file pgen.1003806.s002.tiff]

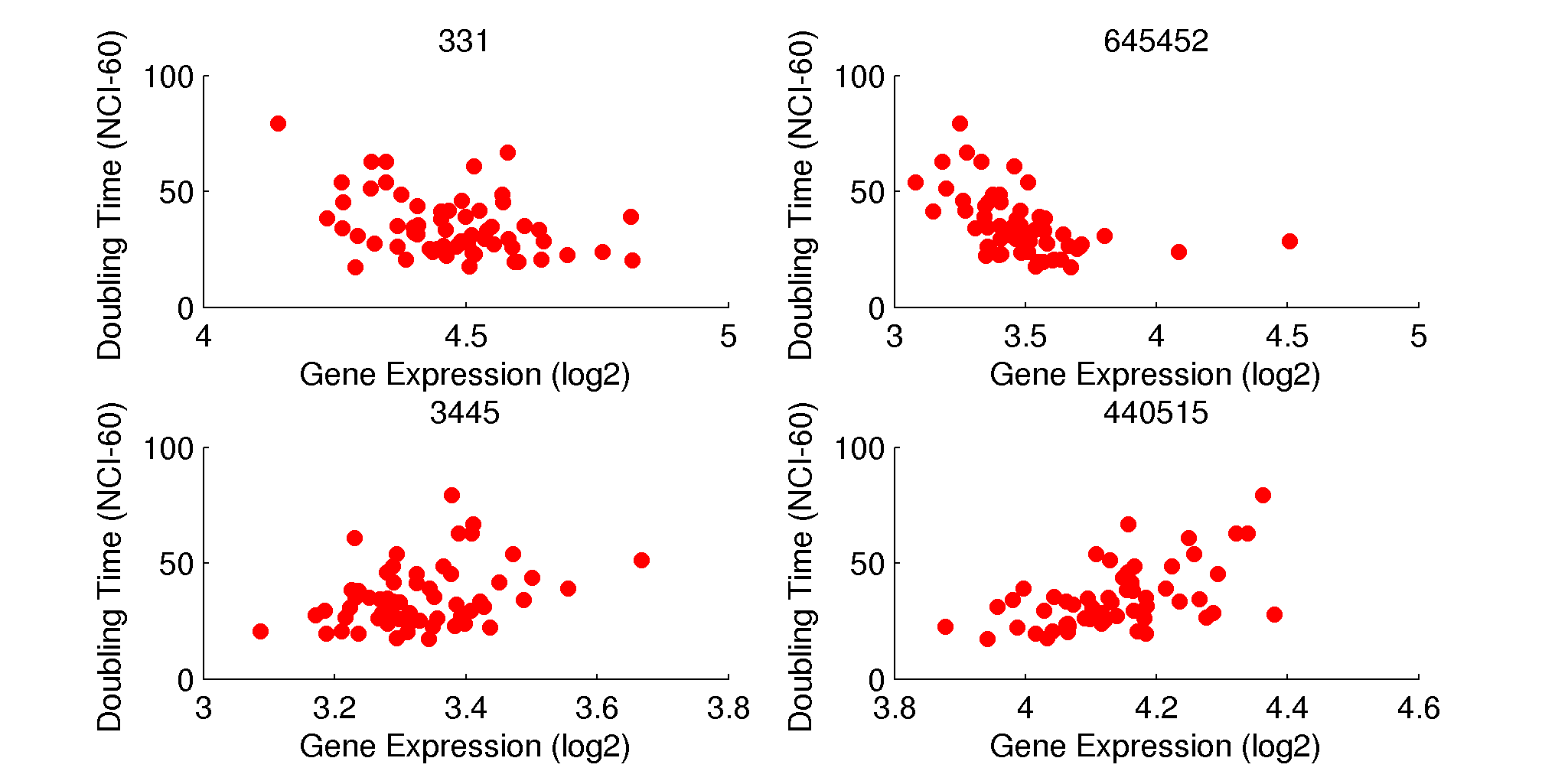

Supplement: Figure S3 — Expression vs. doubling times in the NCI-60 panel for genes with extreme cPI values (slope based). The genes in the top two panels have cPI value above the 99.9 percentile (highest cPI values) while the genes in the bottom two panels have cPI values below the 0.1 percentile (lowest cPI values). The Gene ID (Entrez) for each gene is written above the panel. (TIFF) [file pgen.1003806.s003.tiff]

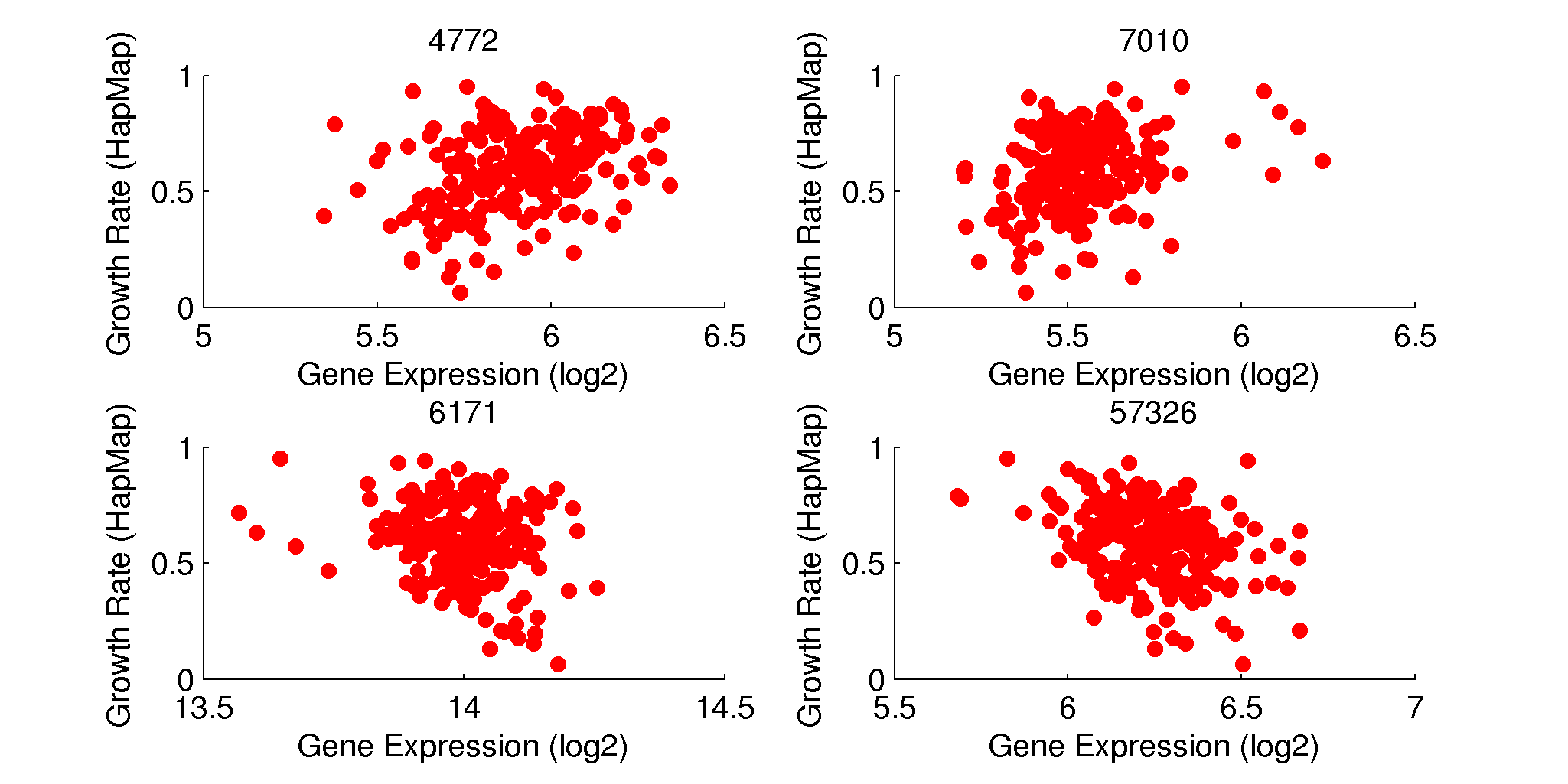

Supplement: Figure S4 — Expression vs. growth rate in the HapMap panel for genes with extreme nPI values (slope based). The genes in the top two panels have cPI value above the 99.9 percentile (highest nPI values) while the genes in the bottom two panels have nPI values below the 0.1 percentile (lowest nPI values). The Gene ID (Entrez) for each gene is written above the panel. (TIFF) [file pgen.1003806.s004.tiff]

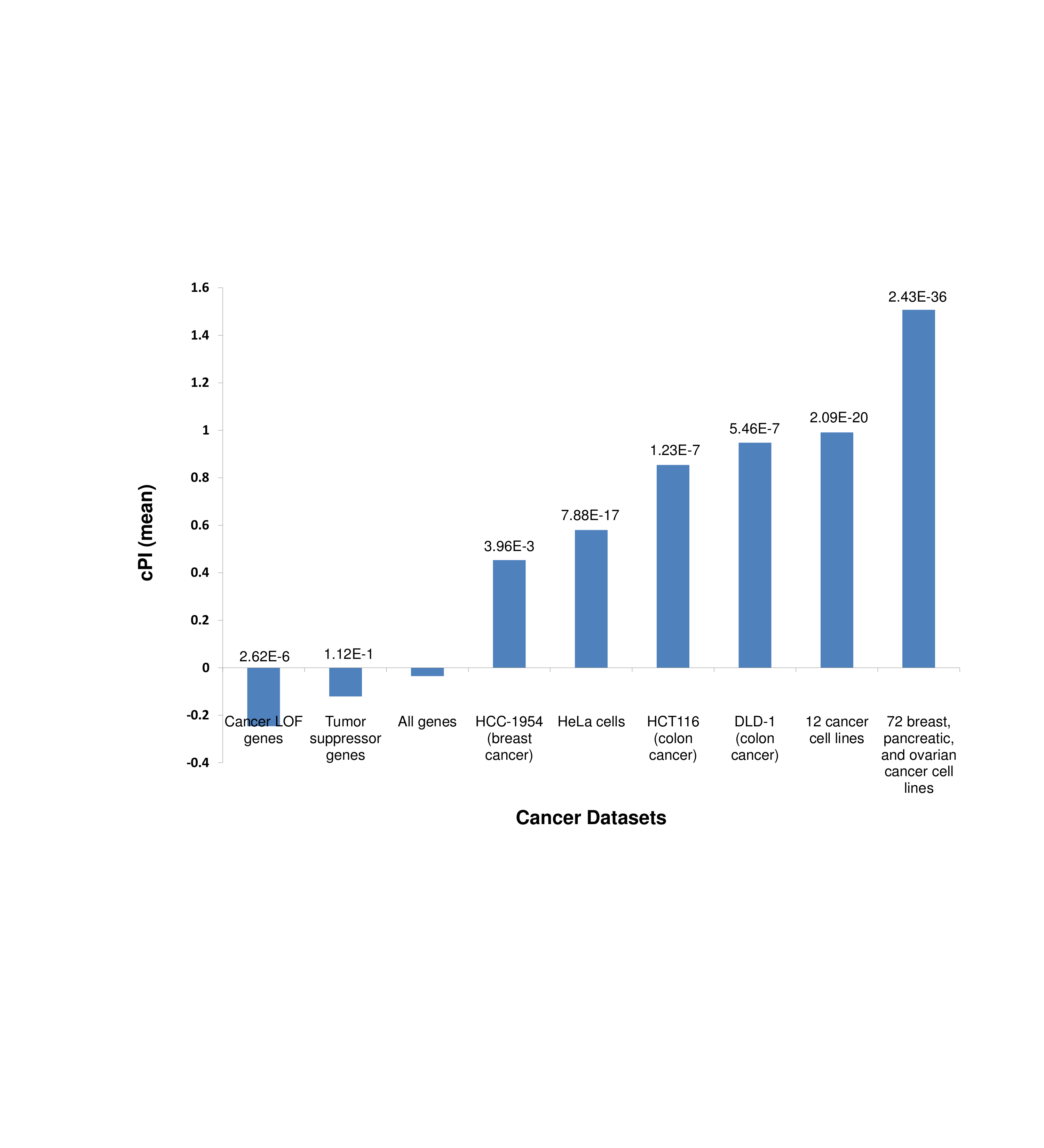

Supplement: Figure S5 — Essential genes and their cPI values (slope based). Using published shRNA screening data we defined sets of genes essential for cancer proliferation in different cancer cell lines (Materials and Methods). Each of these sets exhibits significantly high cPI values as compared to non-essential genes (Wilcoxon rank sum test). In contrast, genes with loss of function (LOF) mutations in various cancers show significantly lower cPI values. The mean cPI value of all genes is also depicted as a reference. (TIF) [file pgen.1003806.s005.tif]

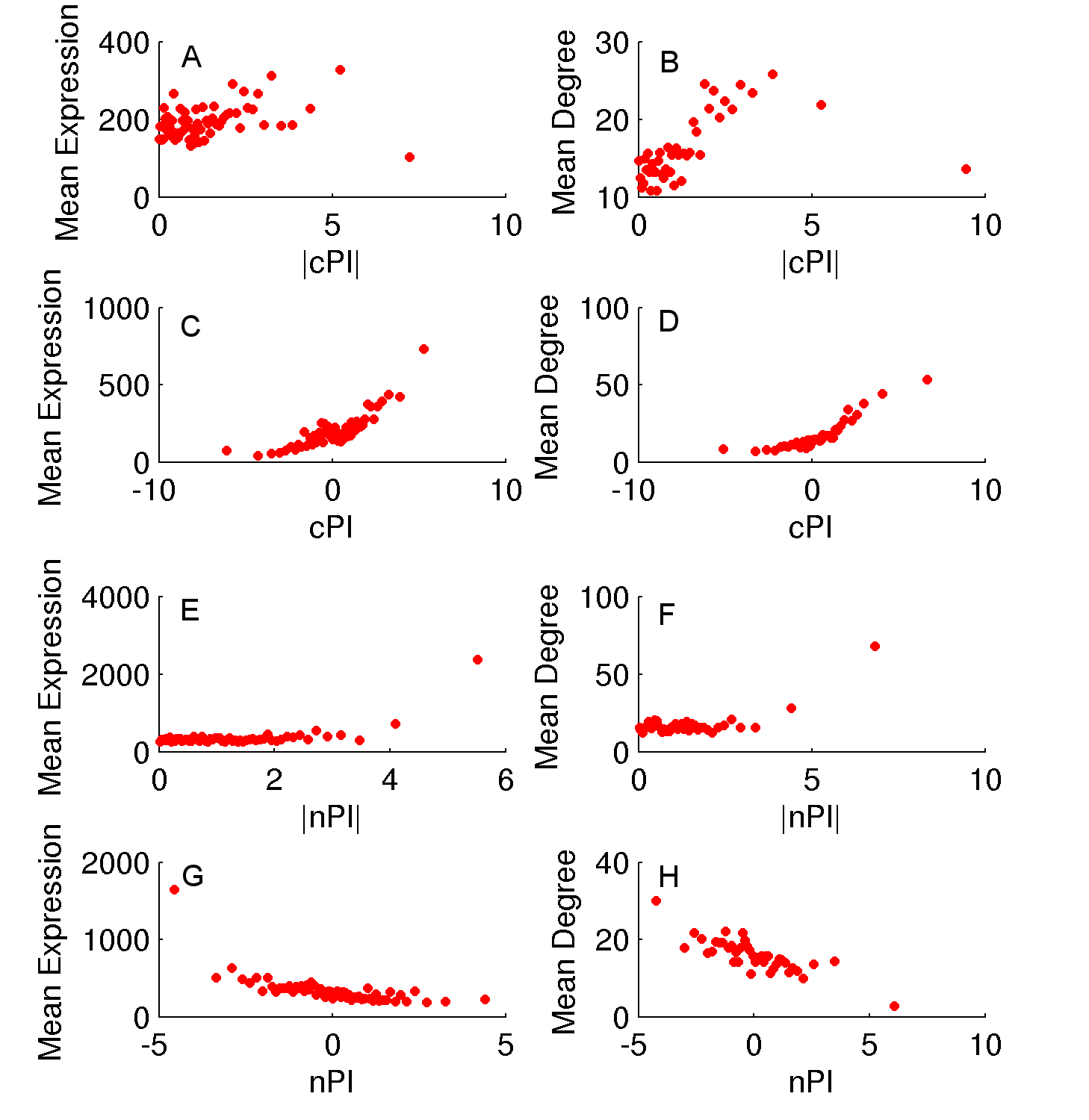

Supplement: Figure S6 — cPI and nPI vs. mean expression and degree in the human PPI network (cPI and nPI are slope-based). Sorting the genes according to their PI measure (nPI, cPI or their absolute values) and binning them (200 genes in a bin), we calculate for each bin the average PI measure, mean expression and degree in the human PPI network. (A) |cPI| vs. mean expression in the NCI-60 panel (R = −0.11, P-value = 2.53e-37 and R = 0.39, P-value = 1.59e-3 for the raw and binned data, respectively). (B) |cPI| vs. degree in the human PPI network (R = 0.04, P-value = 9.54e-5 and R = 0.72, P-value = 3.35e-7 for the raw and binned data, respectively). (C) cPI vs. mean expression in the NCI-60 panel (R = 0.24, P-value≪e-16 and R = 0.79, P-value≪e-16 for the raw and binned data, respectively). (D) cPI vs. degree in the human PPI network (R = 0.18, P-value = 1.32e-61 and R = 0.95, P-value≪e-16 for the raw and binned data, respectively). (E) |nPI| vs. mean expression in 30 adult human tissues (R = 0.01, P-value = 2.11e-1 and R = 0.38, P-value = 2.94e-3 for the raw and binned data, respectively). (F) |nPI| vs. degree in the human PPI network (R = 0.03, P-value = 1.00e-2 and R = 0.20, P-value = 2.15e-1 for the raw and binned data, respectively). (G) nPI vs. mean expression in 30 adult human tissues (R = −0.19, P-value = 3.89e-98 and R = −0.82, P-value≪e-16 for the raw and binned data, respectively). (H) nPI vs. degree in the human PPI network (R = 0.07, P-value = 1.05e-11 and R = −0.80, P-value = 5.15e-9 for the raw and binned data, respectively). (TIFF) [file pgen.1003806.s006.tiff]

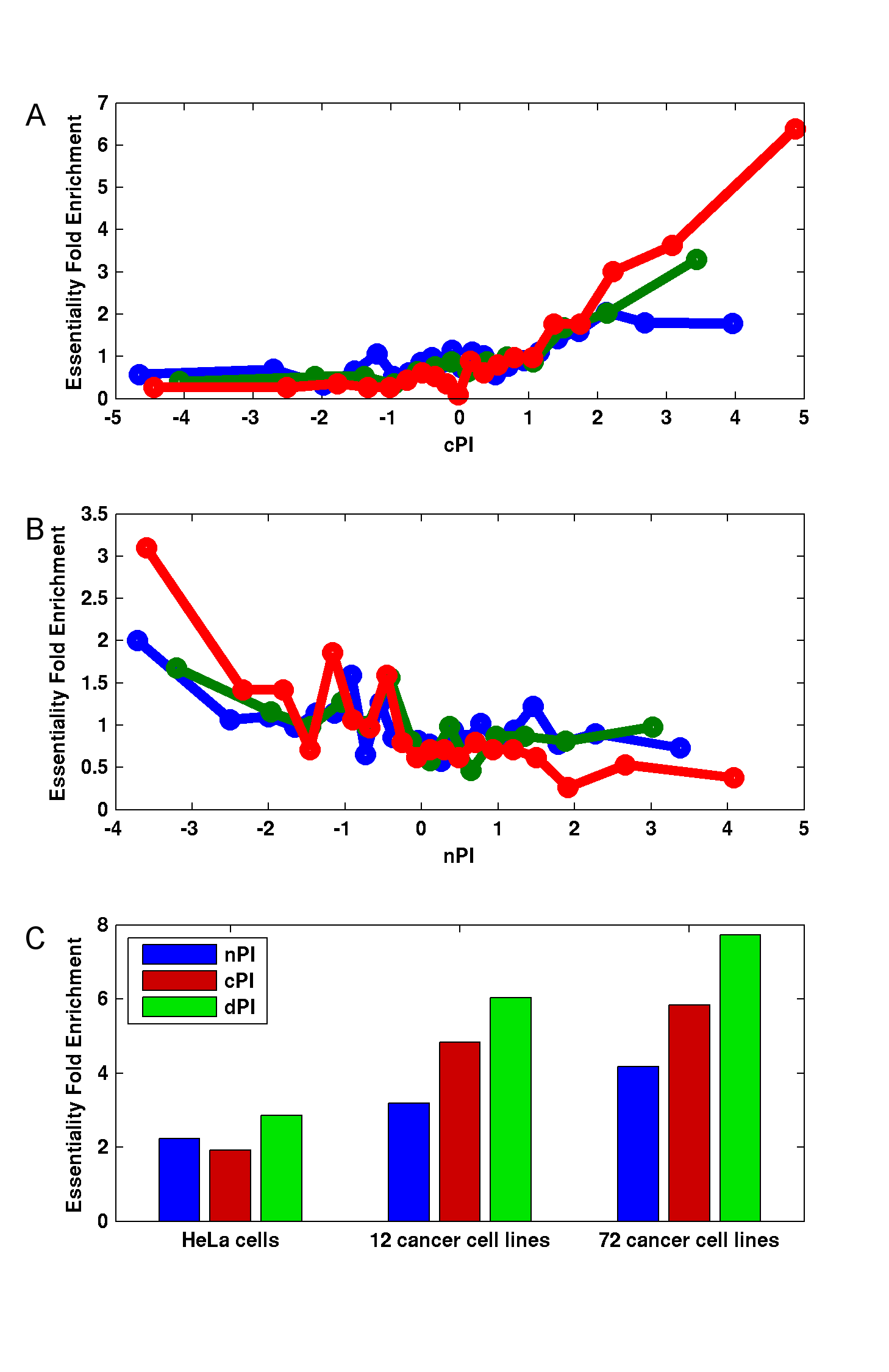

Supplement: Figure S7 — Differential proliferation signatures and cancer gene essentiality. We grouped the genes into bins (200 genes in each bin) according to their (A) cPI and (B) nPI values and measured for each bin the mean measure (cPI or nPI) and the enrichment of the genes in the bin in essential genes in HeLa cells (blue), 12 cancer cell lines (green) and 72 breast, pancreatic, and ovarian cancer cell lines (red). (C) Focusing on a set of 3331 genes with positive cPI and negative nPI values, we defined the top 200 genes for each measure (lowest nPI, highest cPI, highest dPI, correspondingly) and find that dPI shows the highest enrichment in all datasets. Enrichment is significant in all cases (hypergeomteric P-value<e-5). nPI, cPI and dPI measures are slope-based (see main text). (TIFF) [file pgen.1003806.s007.tiff]

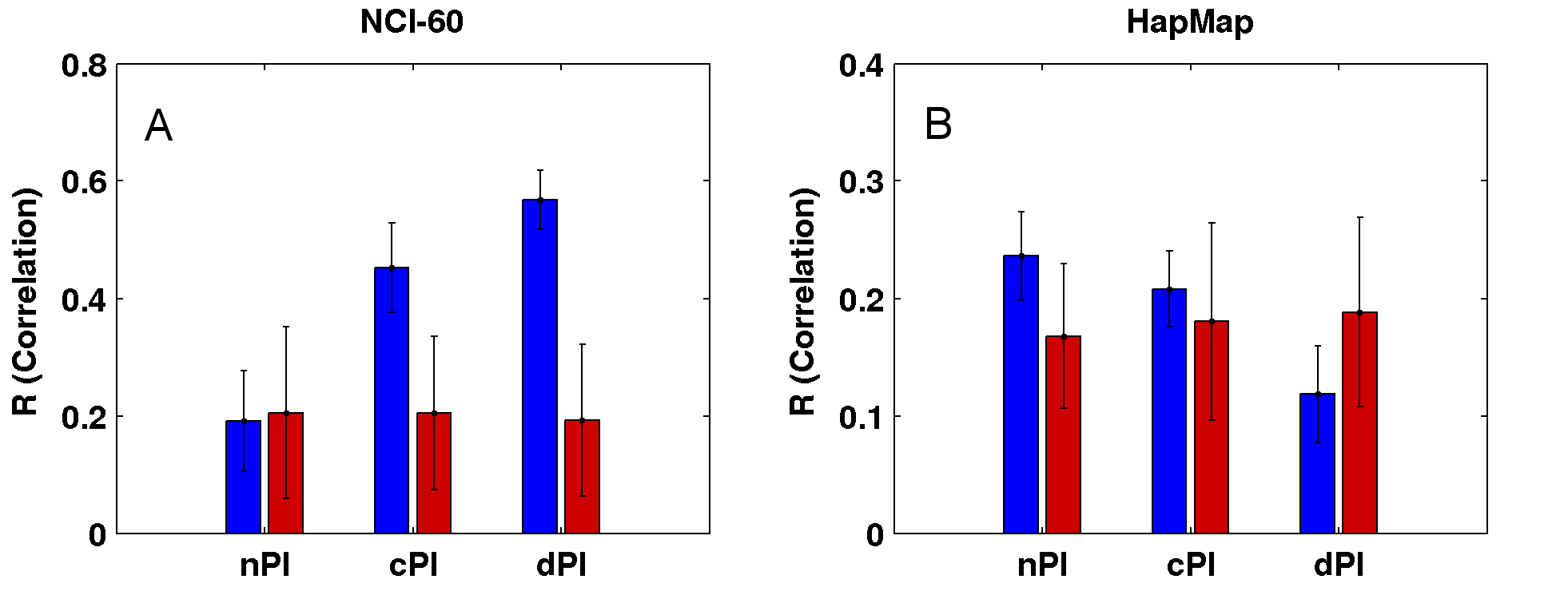

Supplement: Figure S8 — Growth rate predictions of the (A) NCI-60 and (B) HapMap panels. For each signature (nPI, cPI, dPI [slope based]) we compared between the predicted and measured growth rate in the panels. The mean R correlation is presented here for each measure (in blue), in joint with the mean R correlation of equal size random sets of genes (in red). The three measures (nPI, cPI, dPI) are slope-based. (TIFF) [file pgen.1003806.s008.tiff]

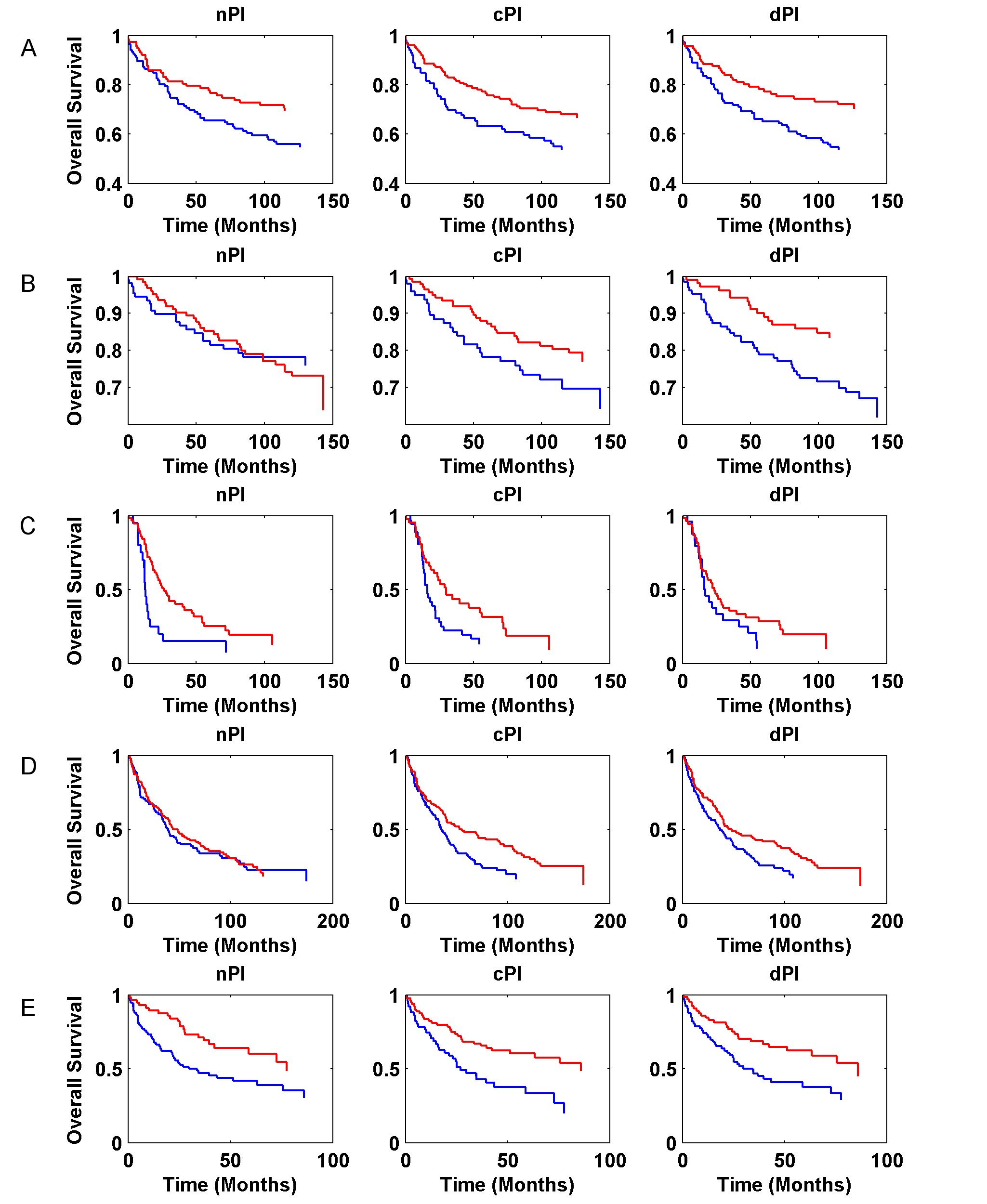

Supplement: Figure S9 — Kaplan-Meier curves for nPI, cPI and dPI signatures (slope-based) in various cancer datasets. (A) breast cancer (Ivshina et al., 249 samples); (B) breast cancer (Miller et al., 236 samples); (C) glioma (Phillips et al., 77 samples); (D) NSCLC (Botling et al., 196 samples). (E) CLL (Chuang et al., 130 samples). The blue and red curves represent lowly and highly predicted proliferating samples, respectively. Additional information is found in Table S5. (TIF) [file pgen.1003806.s009.tif]
