# Supplementary material for: A Genome-Wide Systematic Analysis Reveals Different and Predictive Proliferation Expression Signatures of Cancerous vs. Non-Cancerous Cells
Source: PLoS Genet. 2013 Sep 19;9(9):e1003806. doi: 10.1371/journal.pgen.1003806 (PMC3778010; doi:10.1371/journal.pgen.1003806)
Supplement: Text S1 — Supplementary results (PI measure – correlation vs. slope, replication of the NCI-60 results on a different expression dataset, nPI and nPI-MEM). (PDF) [file pgen.1003806.s016.pdf]

Supplementary Information

A genome-wide systematic analysis reveals different and predictive proliferation expression signatures of cancerous vs. non-cancerous cells

Yedael Y. Waldman[1], Tamar Geiger[2], Eytan Ruppin[1,2]

[1] The Blavatnik School of Computer Science, Tel Aviv University, Tel Aviv 69978, Israel

[2] The Sackler School of Medicine, Tel Aviv University, Tel Aviv 69978, Israel

PI measure – correlation vs. slope

In the current study we define for each gene a measure based on the association between the expression levels of the gene in a set of samples and the growth rates (or doubling times) of these samples. In our analyses we use two measures to quantify this association: the non-parametric Spearman correlation between gene expression and growth rate measurements and the slope of the regression line between gene expression and growth rate measurements (see Materials and Methods). In the main text we describe the results based on the former definition (correlation based). A previous study that analyzed yeast proliferation used a similar measure, which was based on the regression slope between gene expression levels and growth rate measurements [1]. Both measures have their advantages and disadvantages. A measure based on non-parametric correlation coefficient is more robust to differences in scaling of expression levels. On the other hand, a measure based on correlation quantifies how the association between expression and growth rate is similar to a straight line but it does not take into account the magnitude affect of this association, which is reflected in the slope of that line. Yet, in practice the two measures are highly correlated (R=0.89, P-value<<e-16, 12690 genes) and the many of the results reported in the main text (based on correlation) are also replicated when using the slope based measure. This high concordance between the two measures is partially due to the fact that when dealing with high-throughput data (as in this case of gene expression measurements) it is relatively unlikely that there will be a highly significant correlation (either positive or negative) between expression and growth

rate measurements with a small slope, due to the variance of the data. Here we present some of the results when using the slope based measure.
First, we find that essential genes in various cancer cell lines exhibit significantly higher cPI values relative to other genes in these screens. Furthermore, we find that a set of 1352 genes that were found to acquire loss of function mutations in various tumors and are therefore presumed to be enriched with tumor suppressors [2] show significantly lower and negative cPI values, testifying that their decreased functionality may indeed enhance cellular proliferation. Figure S5 summarizes these results.
In addition, we performed functional analysis to find the biological processes that exhibit significant positive or negative cPI values. The results, summarized in Table S3, are very similar to those reported in the main text and in Table S2 (for the correlation based measure). Specifically, cell cycle and processes related to protein production (including translation) are positively associated with proliferation, as well as processes related to oxidative phosphorylation. On the other hand, cellular migration associated processes exhibit significant negative cPI values, in accordance with the "go or grow" dichotomy. The inverse relation between migration and proliferation is further supported by additional datasets. First, we find that sets of genes that are related to cell migration exhibit significantly lower cPI values as compared to other genes: these include genes in the human integrin adhesome [3] (P-value=2.65e-4) and genes whose silencing was previously shown to significantly damage cell migration [4] (empiric P-value=4.01e-2). Second, we assigned for each gene a measure for its relatedness to cell migration based on PubMed papers (Materials and Methods). This measure is significantly negatively correlated with the cPI measure (R=-0.10, P-value=5.64e-30; 12580 genes), in accordance with the "go or grow" dichotomy.

Similar to the results reported in the main text we find notable differences between cancerous and non-cancerous proliferation as reflected by the cPI and nPI measures. First, the two measures show low negative correlation between them (R=-0.05, P-value<<e-16, 12690 genes). In addition, while cPI is positively associated with mean expression in the NCI-60 panel, as well as with the degree of the gene's product in the human protein-protein interaction (PPI) network, nPI is negatively associated with mean expression in 30 normal adult tissues, as well as with the degree in the human

PPI network (Figure S6). In addition, we find that while the cPI measure of a gene is positively correlated with the likelihood of a gene to be essential in cancer cell lines, there is no such association in respect to nPI, where we also see that genes with negative nPI are enriched with essential genes with essential genes. Focusing on a set of 3331 genes that exhibit differential proliferation (genes with positive cPI and negative nPI values) we defined a new measure, the differential Proliferation Index (dPI). We find that this joint measure better predicts cancer gene essentiality as compared to each of the other two measures alone. These results are summarized in Figure S7.

As we show in the main text, cancer proliferation resembles that of microorganisms. Indeed, we find a significant correlation between cPI (slope based) and yeast PI for 1659 orthologous genes (R=0.19, P=6.34e-15), whereas the correlation with nPI and yeast PI is much weaker (R=0.08, P=4.47e-4). Notably, the correlation between nPI and cPI on those 1659 genes is not significant (R=0.02, P-value=0.48). In addition, we looked on sets of genes that were found to be essential in two different yeast species. Indeed, we find that the cPI of their human orthologous genes is significantly higher as compared to other genes (mean cPI=1.18, P-value=1.73e-72 and mean cPI=1.16, P-value=2.40e-89 for *Saccharomyces cerevisiae* (572 genes) and *Schizosaccharomyces pombe* (709 genes) essential genes, respectively; Wilcoxon test). Interestingly, when turning to nPI, we find that these genes have slightly *lower* nPI values as compared to other genes (mean nPI=-0.28, P-value=1.054e-3 and mean nPI=-0.27, P-value=2.09e-4 for *Saccharomyces cerevisiae* and *Schizosaccharomyces pombe* essential genes, respectively; Wilcoxon test).

Replication of the NCI-60 results on a different expression dataset

NCI-60 cell lines are widely used in different labs around the world. Specifically, gene expression measurements of NCI-60 were taken in different platforms. To show the robustness of our results to different expression platforms we repeated the main analysis on a different expression dataset of the NCI-60 panel. The original dataset used in the current study was measured using Affymetrix Human Genome U133A Array (dataset GSE5846 in NCBI's Gene Expression Omnibus repository) while the

additional dataset was measured using Agilent-014850 Whole Human Genome Microarray 4x44K G4112F (dataset GSE29288 in the same repository).

As each cell line had at least two replicates, this dataset (GSE29288) contains 132 samples. For each of the 60 cell lines we averaged expression levels across replicas. Out of 12690 genes used in our analysis, 12293 (97%) also had expression in the Agilent platform and we focused on these genes for our analysis.

Although there is a variation in expression values between the two platforms, the correlation between them is significant. Thus, in the cell line level (i.e., comparing between expression profile of all 12293 genes in the same cell line in the two datasets) we find a mean correlation R=0.73 between the two platforms (P-value<<e-16 in all 60 cell lines). Similar analysis in the gene level (by calculating the correlation of the expression of each gene across all NCI-60 cell lines in the two platforms) achieves a lower mean correlation (R=0.56).

The cPI values based on the different platforms also exhibit a significant correlation: R=0.68 (P-value<<e-16) and R=0.78 (P-value<<e-16; 12293 genes) for the slope based cPI and correlation based cPI, respectively.

Furthermore, we used the cPI values derived from the Agilent platform and looked for GO terms and metabolic pathways that show significant association (either positive or negative) with proliferation. The results are very similar for those obtained using the Affymetrix platform. Specifically, we observe the “go or grow” dichotomy, where terms associated with cell migration exhibit significant negative cPI values. On the other hand, translation associated terms exhibit significant positive cPI values. The results are summarized in Tables S2 and S3.

Taken together, these results show that our analysis is robust to different gene expression measurements platforms (Affymetrix and Agilent).

nPI and nPI-MEM

Our analysis of non-cancerous proliferation is based on 224 lymphoblastoid samples from the HapMap panel, where both gene expression and growth rate measurements are available (Choy et al. [5]). Currently, we lack additional datasets that include both expression and growth rate measurements of non-cancerous cells or cell lines to generalize these results. Nevertheless, we were able to replicate some of the results

using a different measurements based on HapMap samples. Im et al. [6] used a different approach to evaluate the growth rate of HapMap samples, based on growth rate measurements (alamarBlue essay) and a model (mixed effect model averaging, MEM). In addition, they also used gene expression measurements on 176 HapMap samples (136 of them also appear in our dataset) to evaluate the association between growth rate and gene expression of different genes. It should be noted that both gene expression and growth rate measurements were done independently in respect to the measurements we use in the current study. Furthermore, expression measurements were done in two different platforms (Choy et al. used Affymetrix Human Genome U133A Array while the expression data Im et al. used was measured with Affymetrix Human Exon 1.0 ST Array). We used their association measure (nPI-MEM, downloaded from:
http://www.pacdb.org/growthrate/growth.gene.all.data.corrected.txt (beta values)) to see if we can replicate our nPI results and further support them.

First, we examined the correlation between nPI-MEM and nPI and cPI measures. We find that nPI-MEM shows significant correlation to nPI (R=0.16, P-value=1.32e-43 and R=0.176, P-value=6.09e-53 for nPI based on correlation and nPI based on slope, respectively; 7443 genes). The correlation between nPI-MEM and cPI is significant but weaker (R=0.065, P-value=1.80e-8 and R=0.068, P-value=4.34e-9 for cPI based on correlation and cPI based on slope, respectively). Im et al. also defined a set of genes as associated (either positively or negatively) with proliferation, if their FDR P-value was below 0.1. Focusing on this subset of genes, the correlation between nPI and nPI-MEM becomes much stronger (R=0.294, P-value=1.58e-44 and R=0.322, P-value<<e-16 for nPI based on correlation and nPI based on slope, respectively; 2187 genes) and much higher as compared to the correlation between cPI and nPI-MEM on the same set of genes (R=0.118 P-value=3.73e-8 and R=0.136, P-value=2.30e-10 for cPI based on correlation and cPI based on slope, respectively). Similarly, when focusing on those genes with FDR<0.01 (762 genes) the results become even stronger: The correlation between nPI and nPI-MEM is R=0.384, P-value<<e-16 and R=0.40, P-value<<e-16 for nPI based on correlation and nPI based on slope, respectively. The correlation between nPI-MEM and cPI on this set of genes is lower: R=0.155, P-value=1.80e-5 and R=0.163, P-value=6.291e-6 for cPI based on correlation and cPI based on slope, respectively.

Im et al. used DAVID bioinformatics tools [7] to see which cellular processes are enriched with genes associated with proliferation (FDR<0.1). While they did not distinguish in their original analysis between genes that are positively associated with proliferation to those that are negatively associated with proliferation, we repeated their analysis for each group alone. We find that genes that are positively associated with proliferation are enriched with cell cycle related processes (cell cycle, cell division, mitosis etc.). Analysis of genes that are negatively associated with proliferation does not reveal any significant results except to glycoproteins and glycolisations.

In addition to the above enrichment analysis (using DAVID bioinformatics tools) we also repeated our own analysis to see which cellular processes are significantly associated (either positive or negative) with non-cancerous proliferation, similar to the analysis we did for cPI and nPI (as reported in Tabls S2). We find weaker results here, and many of the results obtained by nPI were not replicated here. Nevertheless, we find that oxidative phosphorylation is significantly negatively associated with non-cancerous proliferation, opposed to the results obtained for cPI (and in agreement with the results obtained for nPI, slope based). Table S6 summarizes the results for nPI-MEM analysis.

In conclusion, the results of nPI-MEM are weaker than those obtained for nPI and we discuss in the main text some of the possible explanations for this. Yet, and given its limitations, nPI-MEM analysis reveals that this measure is much more similar to nPI than to cPI, further emphasizing the differences between cancerous and non-cancerous proliferation. We hope that future studies will use more data on both cancerous and non-cancerous proliferation to examine the similarity and differences between them.

References

1. Brauer MJ, Huttenhower C, Airoldi EM, Rosenstein R, Matese JC, et al. (2008) Coordination of growth rate, cell cycle, stress response, and metabolic activity in yeast. Mol Biol Cell 19: 352-367.

2. Solimini NL, Xu Q, Mermel CH, Liang AC, Schlabach MR, et al. (2012) Recurrent hemizygous deletions in cancers may optimize proliferative potential. Science 337: 104-109.

3. Zaidel-Bar R, Itzkovitz S, Ma'ayan A, Iyengar R, Geiger B. (2007) Functional atlas of the integrin adhesome. Nat Cell Biol 9: 858-867.

4. Simpson KJ, Selfors LM, Bui J, Reynolds A, Leake D, et al. (2008) Identification of genes that regulate epithelial cell migration using an siRNA screening approach. Nat Cell Biol 10: 1027-1038.

5. Choy E, Yelensky R, Bonakdar S, Plenge RM, Saxena R, et al. (2008) Genetic analysis of human traits in vitro: Drug response and gene expression in lymphoblastoid cell lines. PLoS genetics 4: e1000287.

6. Im HK, Gamazon ER, Stark AL, Huang RS, Cox NJ, et al. (2012) Mixed effects modeling of proliferation rates in cell-based models: Consequence for pharmacogenomics and cancer. PLoS genetics 8: e1002525.

7. Huang D, Sherman BT, Lempicki RA. (2008) Systematic and integrative analysis of large gene lists using DAVID bioinformatics resources. Nature protocols 4: 44-57.
